# Deciphering the regulatory code of RNA inosine through enzymatic precision mapping and an explainable deep learning model

**DOI:** 10.1038/s41421-026-00930-4

**Published:** 2026-11-04

**Authors:** Shaoqing Han, Jiehua Li, Wei Yang, Qi Wei, Yafen Wang, Xiang Zhou, Xiaocheng Weng

**Affiliations:** 1 College of Chemistry and Molecular Sciences, Key Laboratory of Biomedical Polymers of Ministry of Education, Wuhan University, Wuhan, Hubei China; 2 School of Public Health, Wuhan University, Wuhan, Hubei China; 3 Taikang Center for Life and Medical Sciences, Wuhan University, Wuhan, Hubei China

**Keywords:** Methylation analysis, Bioinformatics

## Abstract

Adenosine-to-inosine (A-to-I) RNA editing is one of the most abundant RNA modifications and is involved in multiple critical biological processes; however, the precise localization of inosine and the mechanisms governing their selective editing remain incompletely understood. Integrating high-accuracy detection technologies with deep learning models can accelerate our understanding of the selective positioning and functional implications of A-to-I editing. To address these challenges, this study simultaneously developed RNA inosine sequencing by endonuclease V (EndoV) and XRN-1 (REX-seq), a highly sensitive and accurate detection method for inosine sites, and Inosine Deep Residual Enhanced Attention Model (InoDREAM), a predictive deep learning framework. REX-seq combines EndoV cleavage specificity with XRN-1 exonuclease activity to enable comprehensive A-to-I editome profiling while maintaining accurate gene expression quantification. Using this method, we identified 590,249 high-confidence inosine sites in HEK293T cells, including 84,645 novel sites. Leveraging the high-confidence A-to-I editing sites identified by REX-seq, we developed InoDREAM, which successfully predicted multiple regulatory proteins that exhibit specific binding affinity to A-to-I edited transcripts while simultaneously identifying disease-associated mutation sites and relevant *cis*-regulatory elements that are functionally linked to RNA editing events. These findings provide novel mechanistic insights into both the molecular regulation of RNA editing and its pathophysiological implications in disease development.

## Introduction

RNA modifications play crucial roles in post-transcriptional regulation, among which adenosine-to-inosine (A-to-I) RNA editing is one of the most common post-transcriptional modifications^1^. This process is catalyzed by the adenosine deaminase acting on RNA (ADAR) enzyme family through the deamination of adenosine to inosine in RNA molecules, significantly increasing transcriptomic diversity and functional complexity^2^. A-to-I RNA editing serves as a crucial post-transcriptional modification that governs essential physiological processes, including neurodevelopment and immune homeostasis. Conversely, the dysregulation of A-to-I RNA editing contributes directly to neurodegenerative diseases, autoimmune disorders, and malignant transformation in cancer^3–7^.

The precise detection of inosine sites is a prerequisite for elucidating their biological functions and associations with disease mechanisms. Because inosine pairs with guanine during reverse transcription, adenosine (A)-to-guanosine (G) base transitions in transcriptome sequencing are typically used to identify A-to-I editing sites^8^. However, this method cannot distinguish between DNA single-nucleotide mutations and sequencing system errors, introducing a large number of false-positive inosine signals^9^; even when genomic DNA sequencing data are integrated, the RNA‒DNA difference (RDD)-based editing site identification method still cannot eliminate the impact of sequence alignment errors^10^. Furthermore, a large number of inosine sites are located in repetitive regions, where short-read sequencing inevitably produces a certain proportion of misalignments^11^. This inherent technical limitation makes accurate identification of A-to-I editing sites one of the most challenging technical problems in current epitranscriptomics research, particularly when studying low-abundance editing events or complex genomic regions, where this issue becomes even more prominent.

Based on chemical reactions and enzymatic conversion, a variety of specific RNA inosine modification detection technologies have been developed, such as the iSeq^12^ method utilizing the enzymatic cleavage activity of ribonuclease T1 (RNase T1), acrylonitrile-based chemical reaction methods such as ICE^13^ and ICE-seq^14^, and endonuclease V (EndoV)-based methods such as EndoVIPER-seq^15^, hEndoV-seq^16^, and Slic-seq^17^. While iSeq can specifically recognize inosine sites, its low enrichment efficiency results in insufficient detection sensitivity; acrylonitrile-based chemical labeling techniques exhibit cross-reactivity with both pseudouridine and uridine, leading to significant off-target effects. Although EndoV-based methods demonstrate high site specificity, they still have notable technical limitations. First, these methods alter the original stoichiometric ratios of RNA editing sites during enrichment, making absolute quantification impossible. Second, the enrichment strategy causes loss of gene expression profile information. More importantly, the periodate oxidation step required for 3′ end processing in these methods introduces severe non-specific RNA degradation, particularly for low-input samples. These inherent shortcomings prevent current methods from simultaneously meeting the dual requirements of highly specific quantitative analysis of transcriptome-wide RNA editing sites and gene expression analysis, greatly limiting the application prospects of RNA editing research in precision medicine.

In recent years, machine learning-based models have advanced rapidly to better elucidate the patterns and mechanisms of RNA A-to-I editing sites. Although GIREMI^18^ enables the genome-independent detection of editing sites, it struggles to distinguish edits overlapping with single nucleotide polymorphisms (SNPs) and exhibits reduced accuracy in non-repetitive genomic regions. Newer methods, such as the DEMINING framework, employ deep neural networks (DNNs) to distinguish A-to-I edits from DNA mutations using sequence context features, achieving high classification accuracy. However, these computational approaches remain constrained by RNA sequencing (RNA-seq) data quality^19^. Third-generation sequencing technologies (e.g., Oxford Nanopore sequencing) detect base modifications by monitoring changes in electrical signals as nucleic acid molecules pass through nanopores. Tools such as Dinopore^20^ and DeepEdit^21^ enable not only RNA editing identification but also resolution of single-molecule co-editing patterns, providing novel insights into the cooperative effects and functional consequences of editing events. However, third-generation sequencing remains limited in clinical scalability due to the complexity of nanopore decoding, reducing accuracy for low-abundance modifications. High input requirements, cost, and sequence-context-dependent signal variations further constrain sensitivity and practicality^22,23^. Alternatively, deep learning-based neural network prediction tools, such as MultiRM^24^ and EditPredict^25^, can predict RNA modifications using only genomic sequences, circumventing the immediate requirement for direct experimental sequencing. These tools integrate reported RNA modification sites and employ deep learning computational methods to achieve high-accuracy, low-cost modification site prediction, but their performance is constrained by the accuracy of upstream modification detection data. For example, EditPredict was trained on 4.67 million computationally derived A-to-I editing sites from the REDIportal database^26^, most of which lack experimental validation; this limitation in training data accuracy restricts the model’s predictive performance and biological interpretability. Currently, most machine learning models for RNA modification prediction prioritize algorithmic performance over in-depth exploration of interpretability and biological significance. This limitation not only hinders breakthroughs in understanding the biological functions of RNA modifications but also significantly impedes their practical applications in precision medicine.

Here, we present a synergistic methodological framework that integrates RNA inosine sequencing by EndoV and XRN-1 (REX-seq), an optimized EndoV-based profiling technique, with Inosine Deep Residual Enhanced Attention Model (InoDREAM), an interpretable deep learning model. By leveraging the specific cleavage capability of EndoV alongside XRN-1 exonuclease properties, REX-seq yields high-accuracy (> 99%) empirical data on RNA editing dynamics. This high-quality dataset, in turn, empowers the InoDREAM framework to decipher the underlying mechanisms of A-to-I editing in regulating the recognition of RNA-binding proteins (RBPs), secondary structure stability, and *cis*-regulatory elements. Together, this integrated REX-seq-InoDREAM axis establishes a unified analytical platform that not only provides a high-precision tool for epitranscriptomics research but also offers new perspectives for understanding the biological functions of RNA editing.

## Results

### Strand-specific truncation in REX-seq mediated by EndoV and XRN-1

**Fig. 1:**
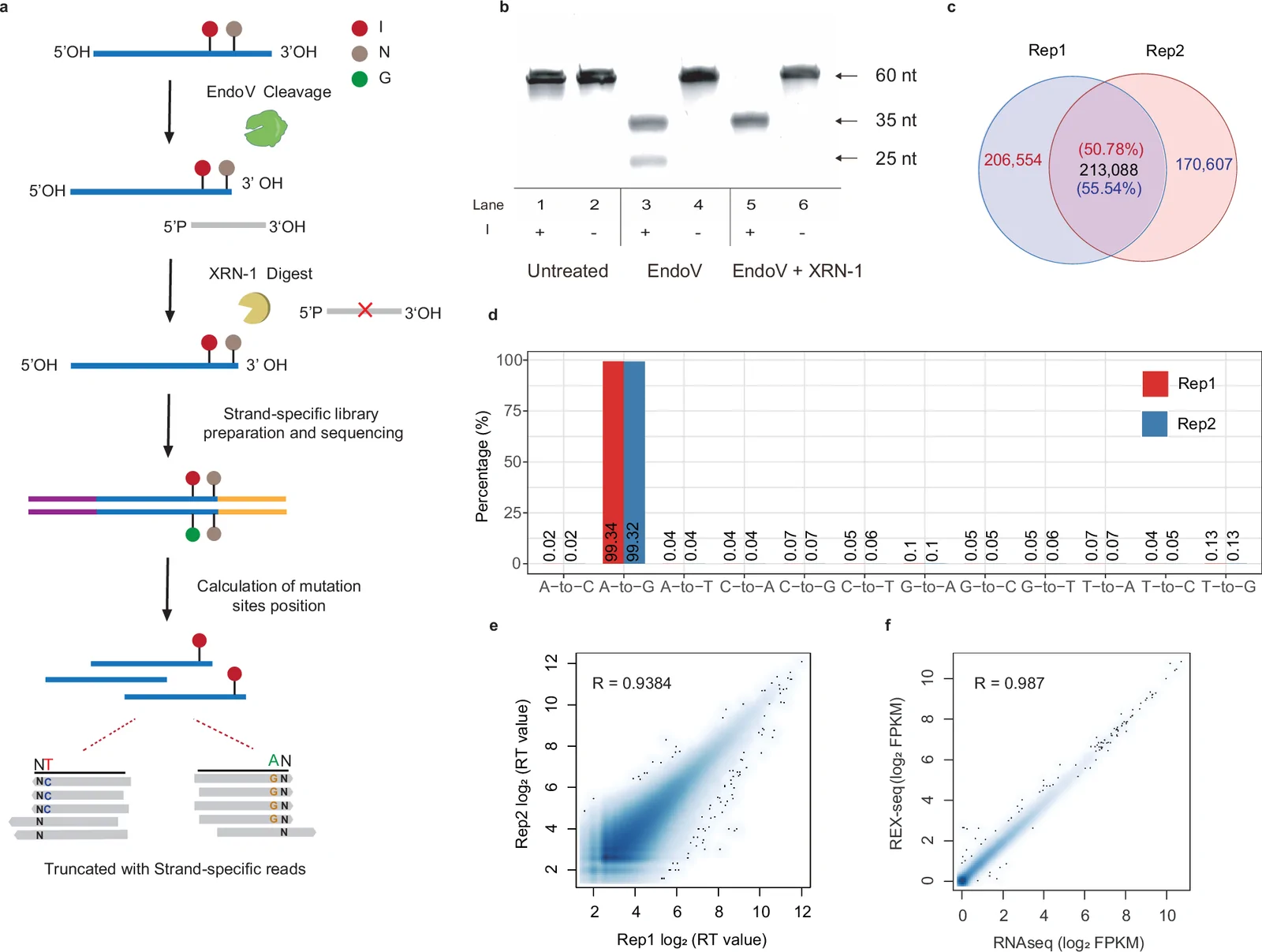
Identification and validation of A-to-I RNA editing sites with REX-seq. **a** Schematic illustration of the single-base resolution of A-to-I detection in RNA by REX-seq. A-to-I editing sites are identified as A-to-G (positive strand) and T-to-C (negative strand) mutations occurring at the 1 bp upstream position from EndoV cleavage sites. **b** In vitro enzymatic validation of EndoV and XRN-1 processing. A 60-nt RNA substrate bearing an inosine at position 34 was treated with EndoV alone or sequentially with EndoV and XRN-1 and analyzed by denaturing PAGE. EndoV cleavage yields two fragments: an upstream 5′-hydroxylated fragment (35 nt, upper) and a downstream 5′-monophosphorylated fragment (25 nt, lower). **c** Overlap of A-to-I RNA editing sites identified by REX-seq between two biological replicates. **d** Mutation profiles at the second base of the reads following EndoV treatment. The bar plot illustrates the frequencies of 12 mutation types occurring at the second nucleotide position in sequencing reads from two independent Endo V-digested samples. **e** Pairwise comparison of truncated reads at A-to-I sites (*r* = 0.94). Scatter plot displays the correlation of normalized truncated read counts between two experimental samples. **f** Replicate correlation of log_2_(FPKM (Fragments Per Kilobase of transcript per Million mapped reads)) values in REX-seq and RNA-seq (*r* = 0.99).

EndoV cleaves at the second phosphodiester bond downstream of inosine, generating 3′-hydroxyl and 5′-phosphate termini^27^. As extensively demonstrated in our prior Slic-seq studies and classic biochemical characterizations, EndoV exhibits stringent specificity for recognizing and cleaving inosine residues within RNA molecules, enabling precise detection of characteristic truncation signatures at inosine loci in RNA-seq profiles. XRN-1 exonuclease, a 5′-monophosphate-dependent 5′→3′ ribonuclease, efficiently degrades RNA fragments bearing 5′-monophosphate termini^28^. Building on this, we developed REX-seq, a novel high-throughput platform that integrates dual-enzyme activity for RNA editing detection. This strategy involves EndoV-mediated recognition and cleavage of inosine residues to generate 5′-monophosphorylated termini, followed by XRN-1-catalyzed degradation of downstream fragments initiated by these termini. We additionally performed an in vitro enzymatic specificity assay using fragmented HEK293T mRNA to confirm that XRN-1 strictly requires a 5′-phosphate terminus for its exonuclease activity (Supplementary Fig. S1). Notably, transcripts that remain uncleaved by EndoV (with native 5′-hydroxyl termini) are resistant to XRN-1 digestion, thereby preserving both intact non-edited transcripts and 5′-terminal fragments of inosine-containing RNAs in sequencing libraries (Fig. 1a). Through this enzymatic approach, sequencing libraries concurrently manifest inosine-specific cleavage signatures and retain complete transcript expression profiles (Supplementary Dataset S1). By selectively removing EndoV-generated 5′-monophosphate fragments, this method prevents systematic quantification errors caused by fragmentation of inosine-containing transcripts (where cleavage of inosine-containing RNAs produces multiple countable fragments and leads to overrepresentation in sequencing libraries), enabling simultaneous base-resolution editing site identification and transcriptome-wide expression profiling. To systematically evaluate the impact of XRN-1-mediated downstream fragment degradation on editing level quantification, we compared editing levels between REX-seq and conventional RNA-seq at 71,666 overlapping Alu sites (Supplementary Fig. S2a–c). Supplementary Fig. S2a shows the distribution of Δ (REX-seq minus RNA-seq editing level) stratified by distance from the 5′ end, revealing a modest positive bias within the first 50 bp (median Δ = +0.0349) that diminishes to near zero beyond 50 bp (median Δ = 0.0000). Supplementary Fig. S2b directly examines the shadowing effect by plotting REX-seq coverage as a function of distance from the 5′ end of each cluster, confirming that XRN-1-mediated degradation reduces downstream coverage, although this effect is localized, and coverage subsequently recovers. Supplementary Fig. S2c depicts the trend of the mean Δ between REX-seq and RNA-seq within clusters, showing a more pronounced bias near the 5′ end that converges to zero near 50 bp and remains stable thereafter, indicating high concordance between REX-seq and RNA-seq editing levels at downstream sites. Collectively, these results demonstrate that REX-seq exhibits only a mild 5′ bias confined to approximately the first 50 bp of clusters (Δ < 0.1) and that editing levels at downstream sites are reliably recovered.

For library construction, we employed a strand-specific sequencing strategy that yields distinct strand-specific distribution patterns of sequencing signals. The cleavage signatures at inosine sites were localized at the +1 position downstream of terminal G nucleotides on the forward strand and appeared at the –1 position upstream of C nucleotides on the reverse strand. This bidirectional cross-validation significantly enhanced the detection accuracy of RNA inosine modifications (Fig. 1a). Next, through in vitro validation using 60-nt synthetic RNA containing inosine modifications, we confirmed that EndoV specifically and efficiently cleaves at inosine sites, whereas XRN-1 selectively degrades only the downstream 5′-phosphorylated fragments generated by EndoV cleavage. RNA strands lacking inosine modifications remained intact with 5′-OH termini, rendering them resistant to XRN-1 digestion (Fig. 1b).

Using this approach, we identified 419,642 and 383,695 inosine sites in two biological replicates of HEK293T cell mRNA, with 213,088 sites common to both replicates (Fig. 1c; Supplementary Dataset S2). Systematic analysis of base substitutions immediately upstream of cleavage sites revealed that A-to-G transitions accounted for 99.32%–99.34% of all mutation types (Fig. 1d), which is consistent with the expected biochemical signature of inosine sites. To further validate the reproducibility of REX-seq, we performed an additional independent experiment in HEK293T cells. Three biological replicates yielded 304,999 consensus editing sites. Comparison with the two original replicates revealed that 123,641 sites were shared across all three datasets, and 62.6% of the newly identified sites overlapped with at least one original replicate (Supplementary Fig. S3a). Mutation profiling of the new dataset revealed 99.29% A-to-G substitutions (Supplementary Fig. S3b), which is consistent with the 99.3% observed initially. These results confirm the high reproducibility and specificity of REX-seq across independent experiments.

To assess the cleavage efficiency of EndoV in the REX-seq workflow, we classified the sequencing reads at each detected inosine site into three categories: cleaved modified reads (truncated A-to-G reads), uncleaved modified reads (full-length A-to-G reads), and background unmodified reads (Supplementary Fig. S4a). Cleavage efficiency was defined as the proportion of cleaved modified reads among all modified reads. Across all 704,328 detected inosine sites, the global cleavage efficiency averaged 89.3%, with uncleaved modified reads accounting for only 10.7% of the total modified signals (Supplementary Fig. S4b, c). This efficiency was consistently maintained across different RNA editing levels and sequencing coverages (Supplementary Fig. S4d, e), indicating that EndoV cleavage operates robustly on the majority of target sites regardless of their editing frequency or expression abundance.

The stoichiometric measurements of inosine sites showed excellent reproducibility between biological replicates (*r* = 0.94; Fig. 1e). Most importantly, the expression levels of inosine-containing genes quantified by REX-seq were highly correlated with those obtained by conventional RNA-seq data (*r* = 0.99; Fig. 1f), confirming the ability of REX-seq to simultaneously achieve transcriptome-wide inosine quantification and gene expression profiling.

### REX-seq vs conventional methods for A-to-I RNA editing detection

**Fig. 2:**
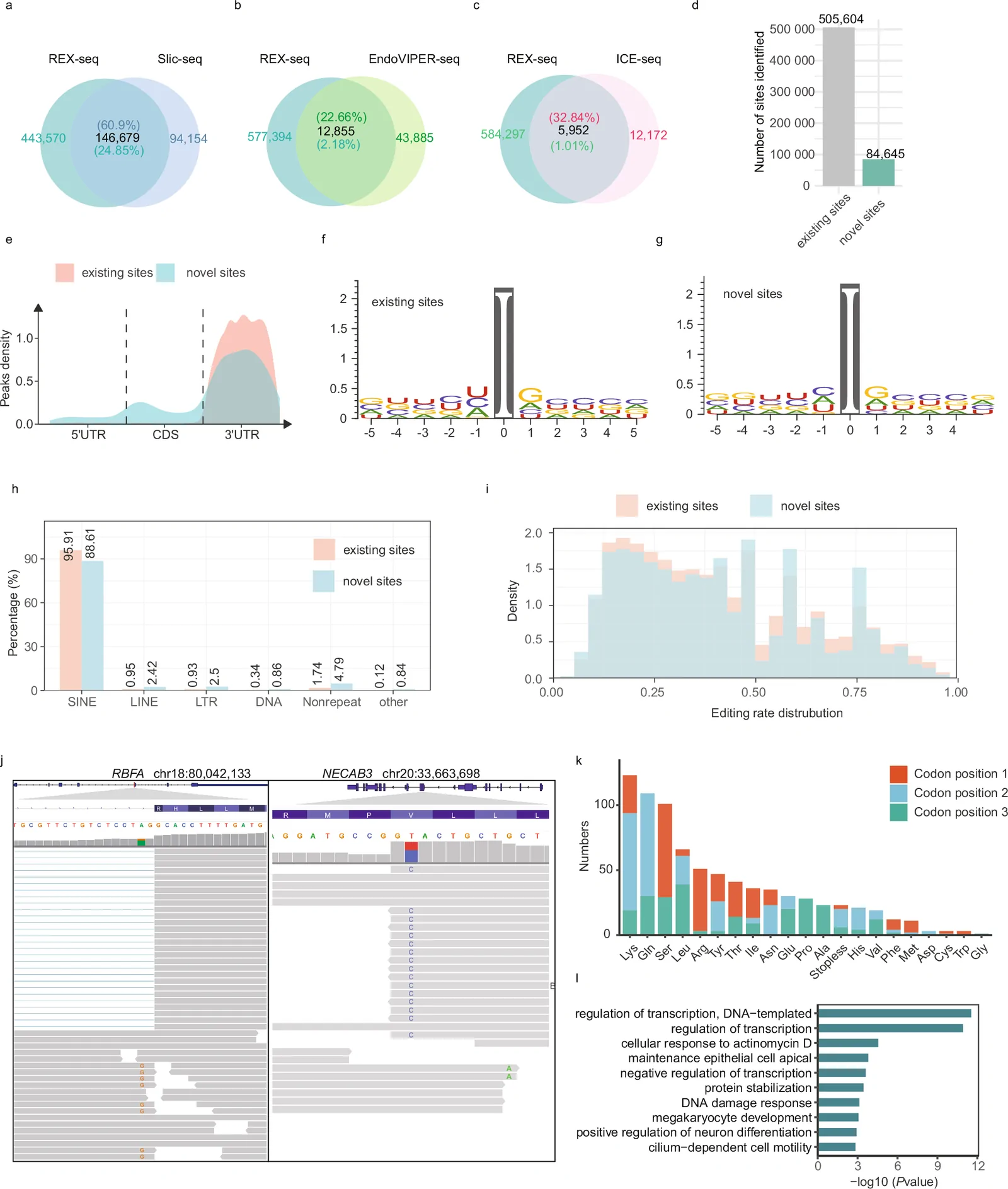
Characterization of A-to-I editing sites detected by REX-seq: methodological comparison, genomic features, and functional profiles. **a** REX-seq and Slic-seq. **b** REX-seq and EndoVIPER-seq. **c** REX-seq and ICE-seq. The numbers of identified editing sites and their overlap proportions are shown in the Venn diagrams. **d** Distribution of A-to-I RNA editing sites in HEK293T cells. **e** Novel A-to-I editing sites show preferential localization in 5′UTRs and CDSs compared with known sites. **f** Motifs of previously reported editing sites detected by REX-seq. **g** Motif analysis of novel editing sites identified by REX-seq. **h** Repeat element distribution of A-to-I editing loci in reported (orange) and novel (light blue) editing sites. **i** Editing level distribution of novel vs reported A-to-I RNA editing sites. **j** Representative REX-seq profiles of inosine editing sites in *RBFA* and *NECAB3* transcripts. **k** Codon editing preference of novel A-to-I RNA editing sites. **l** Functional enrichment analysis of genes with amino acid-altering editing sites.

Comparative analysis of REX-seq and other high-throughput sequencing methods in HEK293T cells revealed that REX-seq-covered sites comprised 60.9% of the Slic-seq dataset (Fig. 2a), with overlap rates of 22.66% against EndoVIPER-seq (Fig. 2b) and 32.8% against ICE-seq (Fig. 2c). To systematically evaluate the performance of REX-seq, we performed a side-by-side comparison with established RNA editing profiling methods, including Slic-seq, EndoVIPER, and ICE-seq, using identical input material from human brain tissues. We first assessed the technical reproducibility of each method. Given the similar biochemical basis between REX-seq and Slic-seq, we compared the reproducibility of the truncated-read signals between biological replicates. Both methods showed high reproducibility, with REX-seq achieving a Pearson correlation coefficient of *r* = 0.969 between biological replicates, which was comparable to that of Slic-seq (*r* = 0.970) (Supplementary Fig. S5a, b). Furthermore, > 99.3% of mutation signals associated with REX-seq truncation sites were A-to-G transitions, supporting the high specificity of inosine-dependent detection (Supplementary Fig. S5c).

We next compared the editing sites identified by each method. REX-seq identified 255,212 RNA editing sites, substantially more than Slic-seq (125,092), EndoVIPER (56,740), and ICE-seq (18,124) did (Supplementary Fig. S6a). Among the identified sites, REX-seq and Slic-seq shared the greatest number of overlapping sites (Supplementary Fig. S6b), which is consistent with their similar detection mechanisms. The cumulative editing frequency distribution further showed that REX-seq detected a broader spectrum of editing events, particularly within the low-frequency range (editing frequency < 0.1), suggesting an enhanced ability to capture low-abundance editing events (Supplementary Fig. S6c). Analysis of replicate-overlapping sites revealed that compared with Slic-seq (25.6%) and EndoVIPER (20.1%), REX-seq achieved a higher replicate sharing rate (35.6%), further demonstrating its robust reproducibility (Supplementary Fig. S6d–f).

We further investigated potential genomic biases among the different methods. Chromosomal distribution analysis revealed highly similar patterns across methods, with all the approaches showing enrichment on chromosomes 1, 7, 17, and 19 (Supplementary Fig. S6g). Analysis of repetitive elements showed that the majority of editing sites identified by REX-seq, Slic-seq, and ICE-seq were located within Alu regions (89.8%, 86.0%, and 95.9%, respectively) (Supplementary Fig. S6h). Finally, we compared editing levels quantified by methods that provide quantitative outputs. Although Slic-seq and EndoVIPER showed higher calculated editing levels (Supplementary Fig. S6i), likely due to differences in library preparation strategies involving targeted enrichment, REX-seq detected editing events across Alu, non-Alu repetitive regions, and non-repetitive regions without targeted enrichment, providing an alternative measurement of transcriptome-wide RNA editing levels. Notably, the low inter-method reproducibility may be primarily attributable to the high abundance and dynamic secondary structural features of inosine modifications in RNA, as RNA secondary structure is known to introduce substantial bias in reverse transcription efficiency^29^ and overall sequencing coverage^30,31^. Critical methodological factors, such as enzymatic cleavage specificity, chemical labeling efficiency, and enrichment strategies, further contribute to these detection discrepancies.

REDIportal is an authoritative RNA editing site database containing more than 15 million human A-to-I editing sites, although most entries lack independent experimental validation^26,32^. By comparing 590,249 inosine sites detected via REX-seq with those in the REDIportal database, we found that 84.2% of the REX-seq sites overlapped with the database records (Supplementary Fig. S7a). This high consistency demonstrates the accuracy of REX-seq. After excluding editing sites identified by existing detection methods and those cataloged in REDIportal, we discovered 84,645 novel A-to-I editing sites (Fig. 2d). Compared with previously reported sites, these novel sites exhibited a greater distribution density in the 5′ untranslated region (UTR) and coding sequence (CDS) regions (Fig. 2e). Motif analysis of the flanking sequences for the novel inosine sites revealed highly consistent base preferences at the +1 position with known sites but a distinct cytosine (C) base preference at the –1 position (Fig. 2f, g). Both novel and known sites displayed similar distributions of repetitive elements: short interspersed nuclear elements (SINEs) predominated (95.91% vs 88.61%), followed by long interspersed nuclear elements (LINEs; 0.95% vs 2.42%), long terminal repeats (LTRs; 0.93% vs 2.5%), and other non-repetitive regions (Fig. 2h). Genomic distribution analysis revealed that both known (76.1%) and novel (78.5%) sites were located primarily in intronic regions (Supplementary Fig. S7b), further supporting the reliability of the novel sites identified with REX-seq. To further validate whether the novel sites identified by REX-seq represent genuine ADAR-mediated A-to-I editing events, we performed REX-seq in wild-type (WT) and ADAR1-null (ADAR1 KO) cell lines (Supplementary Fig. S8a–e). Among the 7610 novel sites with detectable editing signals in WT cells, 7484 (98.3%) showed markedly reduced editing in ADAR1 KO cells, confirming that the vast majority of novel REX-seq sites are dependent on ADAR1 activity (Supplementary Fig. S9a, b).

To independently validate these newly identified editing events, we cross-referenced the 84,645 novel sites against raw sequencing data from an additional HEK293T REX-seq experiment conducted with an independent cell batch. Of these sites, 46,117 (54.5%) were successfully recovered; 40,435 (87.7%) of these validated sites reside within Alu repetitive elements, a hallmark of A-to-I editing. Notably, 40,696 validated sites (88.2%) had low sequencing coverage (< 10 reads), suggesting that their low abundance contributed to their omission in previous catalogs (Supplementary Fig. S10a–c). Together, these results demonstrate that more than half of the newly identified sites are reproducible across independent experiments, further supporting their authenticity as bona fide RNA editing events.

The distribution of editing levels revealed that novel sites were enriched in low-editing-level regions (Fig. 2i), reinforcing our speculation that these sites were likely undetected in prior studies due to their low editing rates. Nucleotide preferences at different editing efficiencies revealed that sites with low editing rates (< 50%) preferred A > C > U at the –1 position, whereas high-editing-rate sites (> 50%) preferred U > C > A (Supplementary Fig. S11a). Across genomic regions, novel inosine sites in the 3′UTR and intronic regions shared similar upstream and downstream sequence preferences, as did those in the 5′UTR and exonic regions (Supplementary Fig. S11b). Although no strictly conserved motifs were identified, the significant sequence biases within ±1 bp of the inosine sites were consistent with those reported in previous research^33^.

Functional characterization of the novel A-to-I editing sites revealed 216 sites possessing critical functional signatures, including 6 key editing sites located at protein splice sites and 210 sites within protein-coding regions. These editing events may regulate gene function by directly altering RNA splicing patterns or protein amino acid sequences. For instance, ribosome binding factor A (RBFA), a highly conserved ribosome assembly factor, acts as a “molecular checkpoint” during the maturation of the small ribosomal subunit (40S)^34^. Our study revealed a previously unreported A-to-I RNA editing site in the *RBFA* pre-mRNA, precisely localized at the 3′ splice site (chr18:80,042,133; Fig. 2j), suggesting that its expression may be post-transcriptionally regulated by ADAR enzymes. This site-specific editing could directly interfere with pre-mRNA splicing, thereby finely modulating ribosome assembly rates. Moreover, RNA editing exerts critical regulatory influence through precisely targeted modifications within protein-coding sequences. Within the coding region of N-terminal EF-hand calcium-binding protein 3 (NECAB3), a neuron-specific calcium-binding protein, we discovered a functional editing site that directly alters the amino acid sequence (Fig. 2j). The *RBFA* gene is encoded on the minus strand; this editing event specifically targets the second base of the antisense UAC codon (encoding tyrosine), converting it to a UGC codon and thereby resulting in a tyrosine-to-cysteine (Tyr-Cys) substitution. Notably, as a core member of the neuronal protein family, loss of NECAB3 function has been confirmed by multiple studies to cause severe developmental defects, including critical phenotypes such as neural tube closure failure and cardiac valve dysmorphogenesis^35^. The newly identified RNA editing sites exhibit significant codon preference, with pronounced enrichment at the second codon position, particularly for lysine (AAG → AIG) and glutamine (CAG → CIG) codons (Fig. 2k). Notably, both showcased novel sites feature characteristic A-to-G transitions 1 bp upstream of the truncation site and exhibit ADAR-dependent editing verified by knockdown data, with one site (chr18:80042133) further validated in an independent human brain dataset, underscoring the high sensitivity of REX-seq for low-abundance events. This editing pattern may represent an evolutionarily conserved targeting mechanism of ADAR enzymes, enabling precise modulation of protein function through specific alterations at key functional sites. Functional annotation of affected amino acid-coding genes revealed that these edits are predominantly associated with transcriptional regulation processes (Fig. 2l). These results demonstrate that the newly discovered RNA editing sites exhibit vital biological functional characteristics. These RNA editing datasets provide substantial augmentation to existing databases such as REDIportal, offering reliable molecular targets for subsequent RNA editing functional studies and disease mechanism exploration.

### InoDREAM accurately predicts inosine sites

**Fig. 3:**
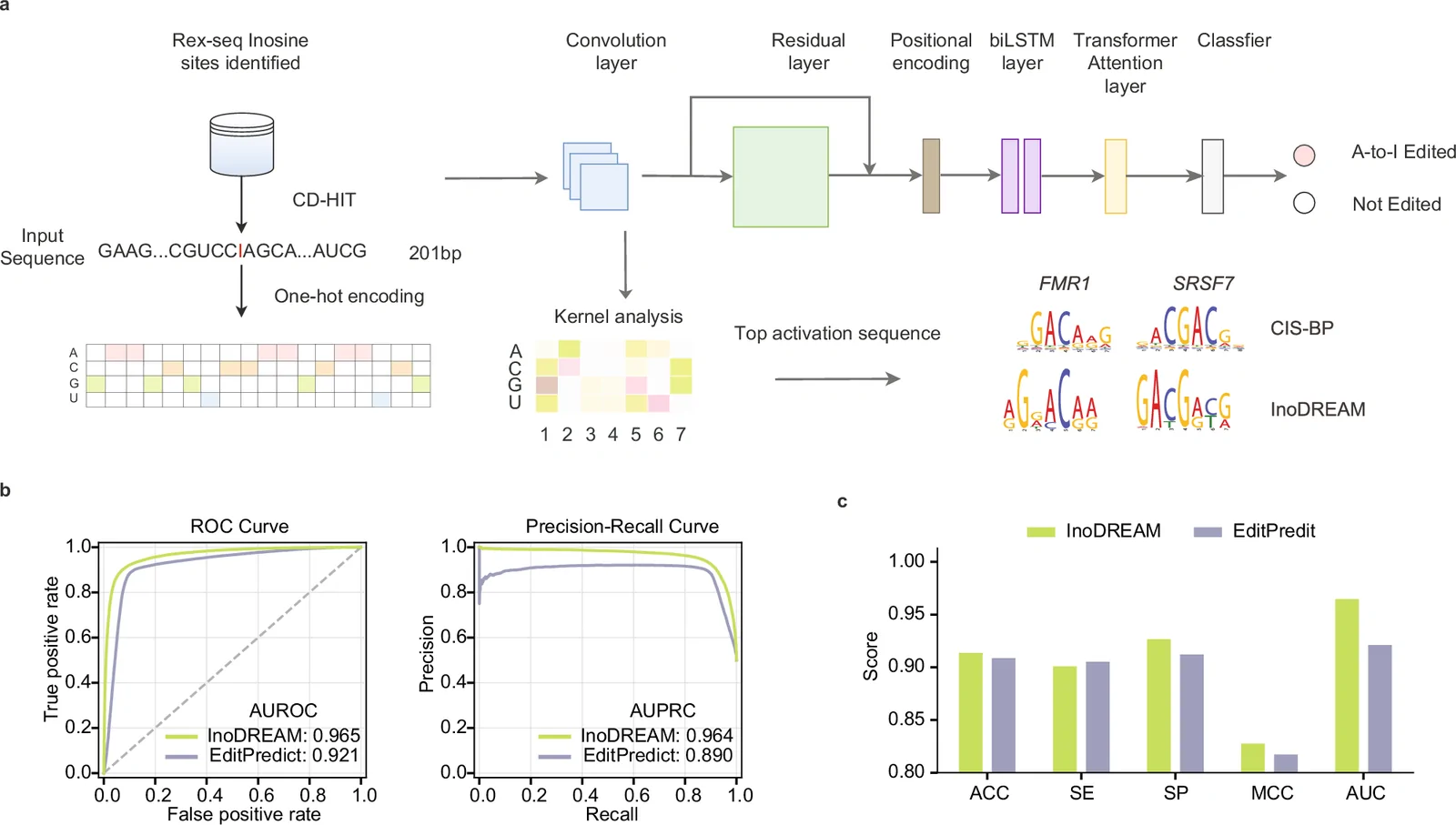
Overview of InoDREAM. **a** Pipeline initiates with one-hot encoding of high-confidence editing sites experimentally validated by REX-seq. Hierarchical feature extraction is performed through convolutional neural networks (CNNs) with residual connections, followed by positional encoding to preserve sequence context. Bidirectional long short-term memory networks then model local sequence dependencies, while transformer attention mechanisms capture global nucleotide interactions, ultimately generating probabilistic predictions of RNA editing events through fully connected layers. **b** ROC curve (left) and precision-recall curve (right) comparing InoDREAM with EditPredict, showing significantly improved performance (AUROC: 0.965 vs 0.921; AUPRC: 0.964 vs 0.890). **c** ACC, SE, SP, MCC, and AUC scores of the InoDREAM and EditPredict methods. ROC, receiver operating characteristic; AUROC, area under the receiver operating characteristic curve; AUPRC, area under the precision-recall curve; ACC, accuracy; SE, sensitivity; SP, specificity; MCC, Matthews correlation coefficient; AUC, area under the curve.

Despite the functional importance of RNA editing, the basis of its site specificity remains unclear. Is RNA editing site specificity determined by a local “code” in flanking sequences, or does it depend on higher-order coordination with *cis*-elements? More intriguingly, how RNA editing modulates RNA–protein interaction networks and their binding preferences awaits elucidation. Leveraging REX-seq high-resolution editome data, we aim to address three fundamental questions: (1) whether editing specificity is determined by proximal sequence features; (2) the potential impact of RNA editing sites on relevant RNA–protein interaction networks; and (3) the existence and characteristic patterns of inosine *cis*-element code. To this end, we developed InoDREAM (Fig. 3a), an interpretable deep learning framework for transcriptome-wide prediction of inosine modification sites. The InoDREAM architecture not only demonstrates powerful feature extraction capabilities, but its built-in interpretability module also uncovers potential sequence determinants, providing a novel computational biology tool to decipher these scientific questions.

To evaluate the performance of the InoDREAM model, we compared it with the existing RNA editing prediction tool EditPredict^25^. Considering the critical impact of high-quality training data on model performance, both methods used the high-confidence RNA editing sites identified in this study as the training set for fair comparison, with validation performed on a hold-out test set. The results demonstrated that InoDREAM significantly outperformed EditPredict in both the area under the receiver operating characteristic curve (AUROC: 0.965) and the average precision (AP: 0.964), compared with EditPredict’s AUROC of 0.921 and AP of 0.890 (Fig. 3b, c). Specifically, performance of the InoDREAM in most metrics achieved an accuracy (ACC) of 0.9137, a specificity of 0.9265, a Matthews correlation coefficient (MCC) of 0.8276, and an area under the curve (AUC) of 0.9647. InoDREAM exhibited significant improvements of 0.56% in ACC, 1.59% in specificity, 1.28% in MCC, 4.73% in AUC, and 8.23% in AP. Next, we additionally compared InoDREAM with two recently published A-to-I editing prediction frameworks: ATTIC^36^ and PreAIS^37^. Within a 51-bp sequence context, InoDREAM achieved an AUC of 0.953 and an AP of 0.950, substantially outperforming ATTIC (AUC = 0.591, AP = 0.550). Within a 101-bp context, InoDREAM achieved an AUC of 0.957 and an AP of 0.959, compared with 0.863 and 0.830 for PreAIS, respectively. These results, together with the comparison against EditPredict presented above, consistently demonstrate the superior performance of InoDREAM for sequence-based editability prediction (Supplementary Fig. S12a, b).

To assess whether the performance of InoDREAM is generalizable beyond HEK293T cells, we trained the model on a large-scale A-to-I editing dataset derived from the REDIportal database, comprising ~1.27 million unique editing sites from a broad range of human tissues and cell types after CD-HIT-based redundancy removal. The model was trained with identical architecture and hyperparameters as the HEK293T model. On the held-out test set, the REDIportal-trained model achieved an AUC of 0.913 and an AP of 0.894 (ACC = 0.858, sensitivity = 0.904, specificity = 0.812; Supplementary Fig. S13a–c), confirming that InoDREAM learns generalizable sequence features that are not limited to a single cell type. The moderate reduction in performance relative to the HEK293T model (AUC = 0.961) is expected, given the substantially greater sequence heterogeneity of the multi-tissue REDIportal dataset than that of the homogeneous HEK293T training distribution. Notably, the REDIportal model maintained strong performance when evaluated on REX-seq data (AUC = 0.942), further demonstrating the transferability of the learned features. These results indicate that InoDREAM effectively captures conserved sequence determinants of A-to-I editing across diverse biological contexts.

To validate the effectiveness of InoDREAM’s architecture, we performed ablation studies on its constituent modules to independently assess each component’s contribution (Supplementary Fig. S14a). The results indicated that InoDREAM surpassed all module-deficient variants in most metrics, except for a marginally higher specificity in the model lacking the attention mechanism. Given the known impact of sequence length on model performance, we assessed model performance under different sequence lengths (Supplementary Fig. S14b). When the sequence lengths were set to 151 bp, 101 bp, and 51 bp, the model’s ACC, AUC, and MCC decreased significantly as the sequences shortened. Although the overall performance improved with longer sequences, the gains plateaued beyond 201 bp, whereas the computational complexity increased. Thus, 201 bp was selected as the optimal length (ACC = 0.9137, AUC = 0.9647, MCC = 0.8276). These results demonstrate InoDREAM’s well-designed architecture and superior performance.

To assess whether InoDREAM’s predictive performance is inflated by the high sequence similarity of Alu repeats, we performed a stratified evaluation on the hold-out test set (74,410 sites) by partitioning sites into Alu repeats, non-Alu repeats, and non-repetitive regions based on RepeatMasker annotation. InoDREAM achieved consistently high performance across all three categories (Supplementary Fig. S15a–c). The robust accuracy observed in non-Alu and non-repetitive regions indicates that the model has learned generalizable editing determinants beyond repetitive sequence identity.

To evaluate whether InoDREAM captures generalizable editing determinants beyond dataset-specific features, we performed independent external validation using datasets from different biological contexts. A cross-method test set was constructed from high-confidence A-to-I editing sites jointly identified by ICE-seq (18,015 sites in the human brain) and EndoVIPER (56,740 sites in the human brain). After 1834 sites overlapping with REX-seq training data were removed to prevent data leakage, 1546 positive sites were retained and paired with an equal number of negative sites randomly sampled from genomic adenosines unreported by any editing detection method. On this hold-out test set, InoDREAM achieved an AUC of 0.9141 (MCC = 0.7037, sensitivity = 0.7374, specificity = 0.9502; Supplementary Fig. S16a–c), indicating that the model maintains robust predictive performance across different biological contexts and captures generalizable sequence features of A-to-I editing.

### InoDREAM provides a protein-binding perspective for deciphering inosine functions

The InoDREAM framework not only delivers high-precision predictive performance but also, more importantly, its interpretable design effectively uncovers underlying sequence determinants (detailed in Materials and Methods). Specifically, we first identified sequence fragments within the first convolutional layer of InoDREAM, where activation values exceeded 90% of the maximum activation (defined as “high-contribution sequences”). By statistically analyzing the position-specific nucleotide frequencies within these sequences, we subsequently transformed the convolutional filters into position weight matrices. Finally, through querying the CIS-BP database^38,39^, we identified potential RNA editing site-binding proteins corresponding to these motifs.

**Fig. 4:**
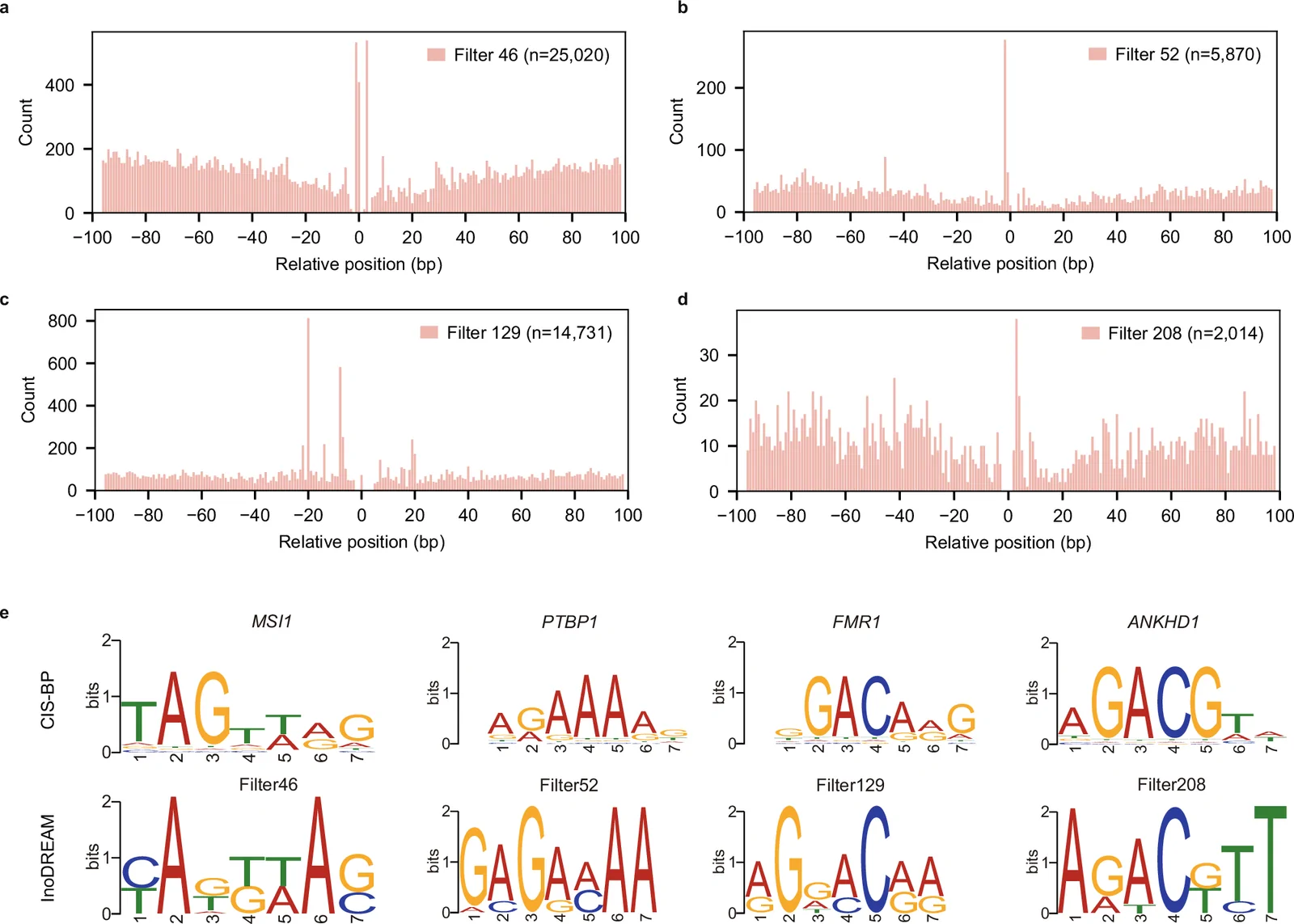
Local sequence motifs captured by InoDREAM on sequences of 201 bp. **a**–**d** Four representative examples showing significant matches between model-learned sequence motifs (**a** Filter 46; **b** Filter 52; **c** Filter 129; **d** Filter 208) and known RBP motifs from the CIS-BP database. Bar plots show the positional distribution frequency of each learned motif across 201-bp sequence windows centered on the inosine site (position 0). The localized enrichment peaks indicate that these sequence motifs are preferentially positioned at specific locations surrounding the editing site rather than being randomly distributed within the sequence context. **e** Sequence motifs derived from the CIS-BP-RNA database (top) are compared with corresponding model-learned motifs (bottom), demonstrating conserved RBP recognition patterns near inosine editing sites.

Interestingly, we observed significant enrichment of multiple key functional elements involved in RNA regulation within the proximal regulatory regions adjacent to inosine editing sites (Fig. 4a–d), including specific binding motifs of RBPs that mediate RNA stability regulation and pre-mRNA splicing (Fig. 4e; Supplementary Fig. S17a, b). For instance, filter 46 learned the binding motif of Musashi RNA binding protein 1 (MSI1), which appears immediately adjacent to inosine sites (Fig. 4a). Previous studies have reported that MSI1 plays dual roles in neural development and tumorigenesis by regulating RNA stability and translation processes^40^. These findings suggest that A-to-I RNA editing may modulate the binding capacity of MSI1 through alterations in its target sequences, thereby influencing its downstream functions. Additionally, binding motifs of other splicing-associated mediators, such as polypyrimidine tract binding protein 1 (PTBP1), fragile X messenger ribonucleoprotein 1 (FMR1), ankyrin repeat and KH domain containing 1 (ANKHD1), serine/arginine-rich splicing factor 7 (SRSF7), and splicing factor 3b subunit 4 (SF3B4), were identified (Fig. 4b–d). These splicing factors play critical roles in RNA processing; for example, ADAR1-mediated editing within the GA-rich sequence of intron 8 in coiled-coil domain-containing 15 (*CCDC15*) significantly enhances the binding affinity of the splicing repressor SRSF7 to this regulatory region, thereby modulating pre-mRNA splicing^41^. Collectively, these important discoveries demonstrate that InoDREAM successfully identified multiple RBPs capable of specifically recognizing inosine-modified sites. The editing events may alter RNA sequence features, affecting the recognition and binding of relevant RBPs and potentially participating in the regulation of pre-mRNA splicing. These findings provide novel insights into the biological functions of A-to-I RNA editing.

To investigate whether the identified RBPs actively modulate ADAR accessibility or function as upstream regulators of the editing process, we performed siRNA-mediated silencing of *MSI1* and *PTBP1* in HEK293T cells. Intriguingly, quantification of A-to-I editing levels at sites overlapping their respective binding motifs revealed only marginal alterations following knockdown (mean Δ = –0.049 for *MSI1*; mean Δ = –0.014 for *PTBP1*). The overall distributions of editing level changes remained tightly centered near zero (Supplementary Fig. S18a–d), indicating that single depletion of these RBPs does not induce widespread, baseline alterations in global RNA editing. Consequently, these motif enrichment profiles are best interpreted as candidate regulatory features and potential downstream pathways associated with edited transcripts rather than as definitive evidence of direct, upstream causal regulation.

In addition to site-specific RBPs, we identified multiple splicing-associated proteins with broad binding properties whose characteristic motifs showed dispersed distribution patterns both at RNA editing sites and in flanking regions (Supplementary Fig. S17c–e), suggesting their potential involvement in RNA editing regulation through non-site-specific mechanisms. For instance, we observed widespread distribution of binding motifs from the RNA processing regulator cytoplasmic polyadenylation element binding protein 2 (CPEB2), spliceosome-associated protein spliceosome-associated factor 3 (SART3), core splicing factor U2 small nuclear RNA auxiliary factor 2 (U2AF2), and synaptic RNA-binding protein Synaptotagmin binding cytoplasmic RNA-interacting protein (SYNCRIP) across editing site regions.

In summary, InoDREAM’s predictions successfully identified multiple RBPs with specific affinity for inosine sites. Based on a comprehensive motif analysis of both site-sensitive and site-insensitive RNA-editing-associated proteins, our results suggest that A-to-I editing may dynamically modulate local sequence features to selectively regulate RNA–protein interactions. These model-derived findings are consistent with established RNA editing functions^42^, providing crucial insights into the molecular mechanisms of post-transcriptional regulatory networks.

### InoDREAM characterizes *cis*-elements governing inosine deposition

**Fig. 5:**
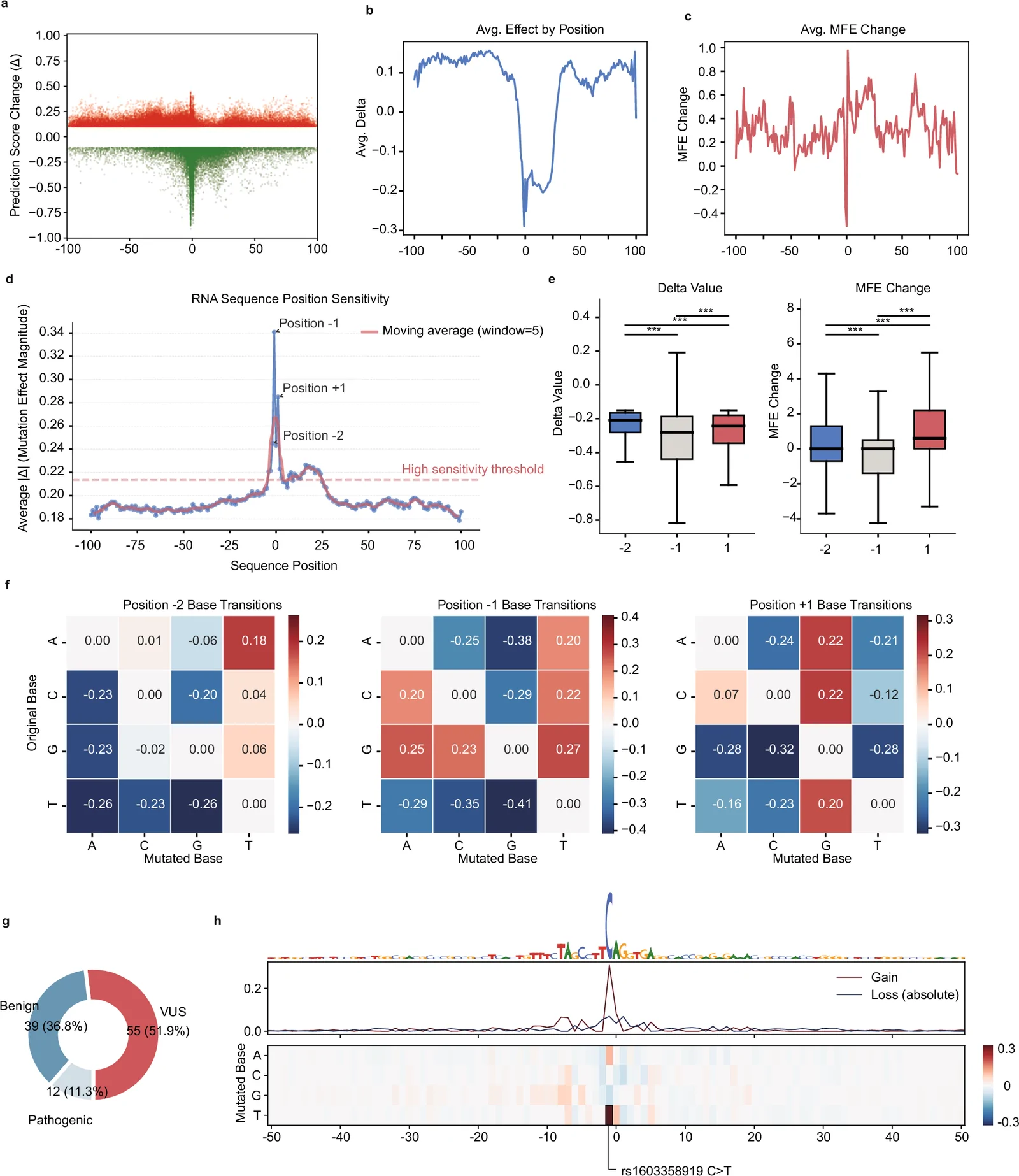
*Cis*-regulatory elements controlling inosine deposition are predominantly located within 40-nt surrounding editing sites. **a** Positional plot of ΔProbability (cutoff = 0.1) within 201-bp windows centered on inosine sites. Single-nucleotide saturation mutagenesis was performed for each position (–100 to +100) around the inosine sites by substitution with the three alternative nucleotides. The changes in inosine probability (ΔProbability) were calculated using the InoDREAM model. **b** The mean ΔProbability profile reveals position-dependent effects on inosine deposition. **c** Quantification of mutation-induced RNA structural perturbations (ΔMEF) within inosine-containing regions. **d** Positional sensitivity analysis of mutational effects (ΔProbability) on inosine. **e** Distribution of InoDREAM-predicted ΔProbability values for all possible nucleotide substitutions at the –2, –1, and +1 positions relative to inosine sites (left). Corresponding changes in RNA structural stability (ΔG, kcal/mol) for mutations at these positions (right) (*n* = 20,344 for –2 positions; *n* = 112,161 for –1 positions; *n* = 59,739 for +1 positions). The medians and interquartile ranges are presented in the box plot. The *P* values were calculated by the one-sided Student’s *t*-test (significance: **P < 0.01, ***P < 0.001). **f** Position-specific effects of base transitions on RNA editing predictions. **g** Pie chart showing the distribution of ΔProbability caused by SNVs classified by ClinVar clinical significance. VUS means uncertain significance SNVs, benign means benign and likely benign SNVs, and pathogenic means pathogenic and likely pathogenic SNVs. **h** Heatmap displaying the ΔProbability for each mutation event. Line plots display the minimum (loss) and maximum (gain) changes among the possible substitutions. Sequence logos highlight the most significant regulatory motifs in the region.

Although deep learning models can accurately predict RNA modification sites, they fail to elucidate the underlying mechanistic principles. Similar to breaking an encoded message, we achieve nucleotide-level positional resolution, but the higher-order regulatory logic remains elusive. To systematically decipher the *cis*-regulatory principles governing RNA editing, we performed comprehensive saturation mutagenesis spanning ±100 nucleotides flanking inosine sites, followed by quantitative assessment of editing efficiency alterations using InoDREAM. To computationally define the functional boundary of these *cis*-regulatory elements, we executed in silico single-nucleotide saturation mutagenesis across a 201-bp window flanking the inosine sites using our InoDREAM model. Specifically, each native nucleotide from positions –100 to +100 was systematically substituted with the three alternative bases, followed by a quantitative evaluation of the resulting shifts in model-predicted editing probabilities (Fig. 5a). Strikingly, our analysis revealed a highly constrained 40-nt regulatory core (–10 to +30 relative to the editing site) exhibiting a maximal density of functional *cis*-elements (Fig. 5b). Single-nucleotide substitutions within this hotspot region induced statistically significant perturbations in editing probability (|ΔProbability| > 0.1), suggesting that this sequence segment constitutes a critical determinant for precise modulation of ADAR-mediated editing.

Considering that RNA modification deposition may be regulated by its secondary structure, we further analyzed the impact of RNA secondary structural changes induced by saturation mutations on inosine deposition. The results revealed that mutations located two nucleotides upstream and downstream of the inosine site exerted significantly opposite effects on RNA structure, whereas mutations in other regions had relatively minor impacts (Fig. 5c). These findings suggest that mutations at these two positions may influence inosine deposition by altering the secondary structure near the inosine site. To systematically evaluate the sensitivity characteristics of positional effects in RNA sequence mutations, we calculated the mean absolute values of predicted changes for all possible mutations at each flanking sequence position of the inosine site based on the InoDREAM model. We identified three highly sensitive positions that significantly exceeded the average sensitivity level, namely, the –1, +1, and –2 positions (Fig. 5d). Mutations at these three positions all reduced the model’s predicted values, but the –1 and +1 positions exhibited opposing trends: mutations at the –1 position decreased the minimum free energy (ΔMFE > –0.4 kcal/mol) on average, indicating enhanced structural stability, whereas mutations at the –2 and +1 positions increased the minimum free energy (ΔMFE ≈ +1.0 kcal/mol), suggesting reduced structural stability (Fig. 5c, e). Given the potential variations among different mutation types, we further analyzed mutations at these three positions and found that mutations to G at the –2 and –1 positions decreased the RNA editing prediction values, whereas mutations to G at the +1 position significantly increased the RNA prediction results. Conversely, mutations to T at the –2 and –1 positions elevated the model’s predictions, whereas mutations to T at the +1 position reduced the RNA editing prediction values (Fig. 5f). These opposing prediction outcomes arising from mutations in the upstream and downstream bases of the inosine site provide critical insights for understanding the *cis*-regulatory mechanisms of RNA editing. Notably, a distal signal around the +25 position also systematically exceeded the sensitivity threshold, potentially reflecting long-range RNA structural features or unconventional distal regulation that warrants future experimental exploration.

Previous studies have reported that a large number of single-nucleotide variants (SNVs) are associated with alterations in inosine editing^7^. Although existing studies have indicated that abnormal regulation of inosine is related to various diseases, the mechanism by which nucleotide variations affect inosine deposition remains unclear. To explore the potential association between SNVs and the regulation of inosine deposition, we systematically screened SNVs located within 200 nucleotides flanking inosine sites from the ClinVar database. By calculating the impact of SNVs on inosine deposition, we found that a subset of SNVs can significantly increase or decrease the probability of inosine deposition (|ΔProbability| ≥ 0.1) (Fig. 5g), including 12 pathogenic mutation sites, 39 likely pathogenic sites, and 55 variants of unknown pathogenicity. We next examined the genomic and repeat context of the variant-affected sites to clarify their functional relevance. Pathogenic sites were predominantly in protein-coding exons (67%, 8/12), with the remaining 4 sites in intronic regions. Variants of uncertain significance (VUS) also showed enrichment in coding and regulatory regions (62% exons, 29% UTRs), whereas benign sites were distributed across intronic regions (54%), coding exons (28%), and UTRs (15%). In terms of repeat context (Supplementary Fig. S19a, b), both pathogenic sites (92%, 11/12) and VUS (78%, 43/55) were predominantly in non-Alu regions, whereas benign sites were less common in non-Alu regions (41%, 16/39). These distinct distributions indicate that clinically pathogenic mutations preferentially disrupt highly specific, non-repetitive editing events in coding or regulatory sequences rather than background editing within deep intronic elements.

We present four typical examples of SNVs that affect inosine deposition. In the *FLNA* gene, rs1603358919 (C-to-T mutation) disrupts the C base in the sequence immediately preceding the inosine site, and InoDREAM predicts that it increases the inosine deposition effect by 0.3 (Fig. 5h). Studies have reported that this gene can cause neurons to fail to reach the cerebral cortex and accumulate abnormally around the ventricles, thereby triggering symptoms such as epilepsy and intellectual disability. Mutations in the *FLNA* gene have also been found to be closely associated with focal cortical dysplasia, tuberous sclerosis complex, and autism spectrum disorder^43^. These findings suggest that the pathogenic single-nucleotide mutation rs1603358919 may alter the inosine modification pattern of *FLNA* gene transcripts, thereby affecting their RNA function and ultimately promoting disease development and progression. rs1176643762 in the *TTC7A* gene (Supplementary Fig. S20a) is an A-to-G mutation occurring at the position immediately preceding the inosine site, resulting in a 0.26 reduction in the inosine deposition effect. This gene has been shown to maintain normal immune and intestinal homeostasis by regulating chromatin spatial structure and gene expression networks^44^ and is annotated as “likely pathogenic” in ClinVar. The rs530900959 mutation in the *BAG2* gene is an A-to-G mutation located 22 bp downstream of the inosine site, leading to a 0.19 decrease in the inosine deposition effect (Supplementary Fig. S20b). The *BAG2* gene is involved in the pathogenesis of various diseases, including cancer and neurodegenerative diseases^45^, and this variant is annotated as a variant of unknown significance in ClinVar.

Interestingly, the InoDREAM model can not only predict the regulatory effect of SNVs on inosine deposition but also identify the impact of key conserved sites on inosine deposition. For instance, the neurodevelopmental protein NDE1 plays an important role in neuronal migration. The C base located 16 bp upstream of *NDE1* plays a critical role in inosine deposition, and the indel rs546308029 occurring at this position results in a 0.41 reduction in the inosine deposition effect (Supplementary Fig. S20c). Mutations in the *NDE1* gene (which is involved in the regulation of cytoplasmic dynein) can cause diseases characterized by severe microcephaly and severe cognitive impairment^46^. rs546308029 also shows likely pathogenicity in the ClinVar database.

Annotating the disease relevance of massive numbers of SNVs represents a major challenge in current genomic medicine. The value of InoDREAM lies in its ability to independently assess the impact of these variations on inosine deposition. Currently, the ClinVar database primarily includes pathogenic SNVs that cause functional abnormalities by directly affecting protein-coding sequences (such as missense and nonsense mutations), and the pathogenic mechanisms of such variations are relatively clear. This study innovatively provides a new dimension of functional annotation for these known pathogenic SNVs from the perspective of A-to-I RNA editing regulation. As the mechanism of action of inosine modification in disease occurrence becomes increasingly clear, it is expected that ClinVar will gradually include genetic variations that cause diseases solely by regulating RNA modifications (such as altering inosine deposition patterns) in the future. The InoDREAM model will provide important technical support for the development of this emerging research field, supplement the existing disease mutation annotation system centered on coding regions, and offer an innovative perspective for understanding RNA editing-related diseases.

### Characteristics of *cis*-elements regulating inosine deposition in the –10 nt to +30 nt region of inosine sites

**Fig. 6:**
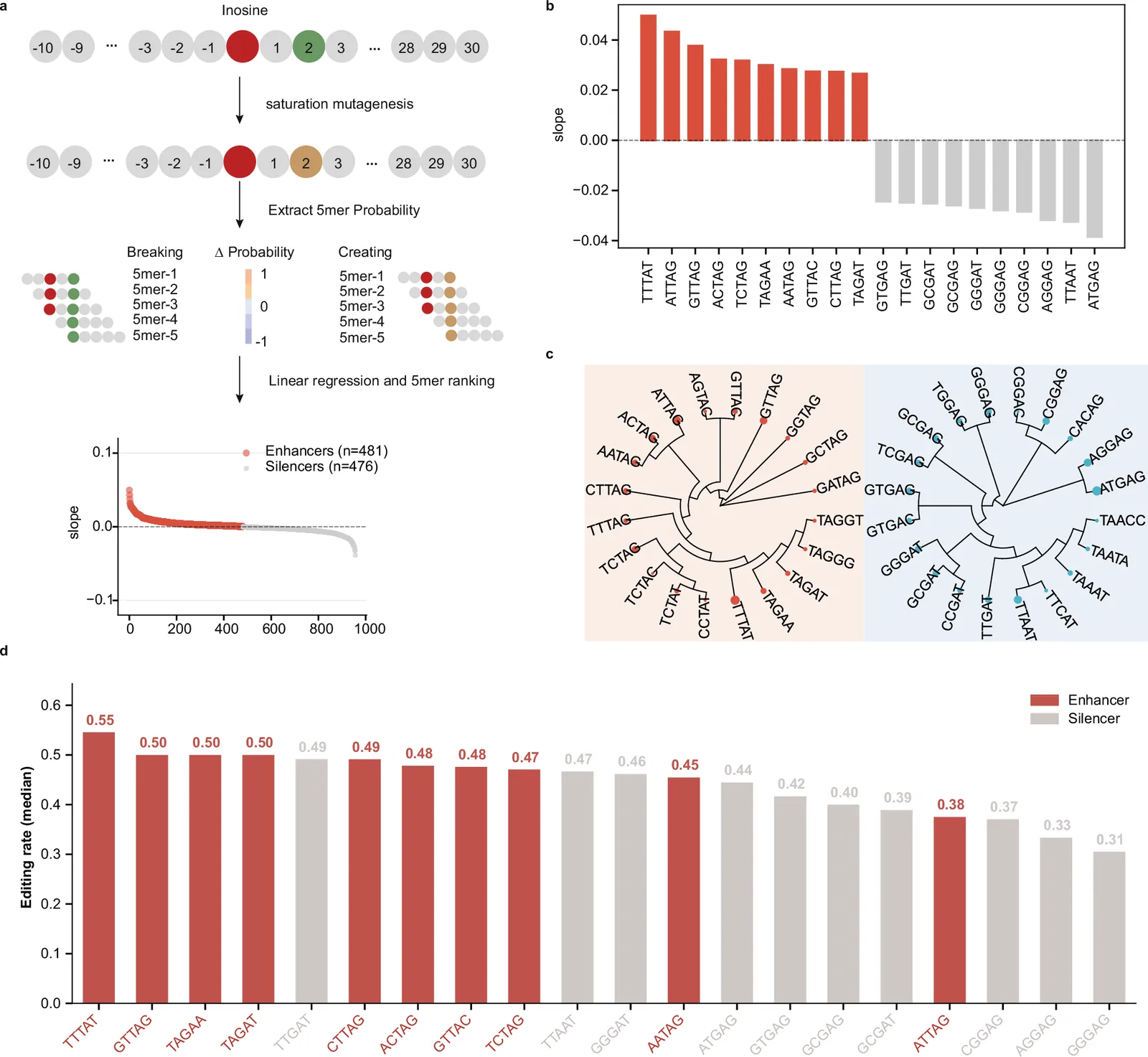
Predicted enhancer and silencer elements within 40-nt inosine editing sites. **a** Systematic and quantitative determination of all *cis*-element pentamer effects on inosine deposition using linear regression. **b** Bar plot showing the effect values of the top 10 enhancer and top 10 silencer 5-mers. **c** Hierarchical clustering of the top 20 regulatory motifs. Enhancer motifs (red) show partial TAG consensus, while silencer motifs (gray) are characterized by GG/AG sequences. **d** Editing level distribution of inosine sites in the top 10 enhancer and silencer sequences. The distribution of inosine editing levels shows preferential localization of enhancer motifs (red) in high-editing regions compared with silencer motifs (gray).

Given that mutations significantly altering inosine deposition (with |ΔProbability| > 0.1) are predominantly enriched within the 40-nt region of inosine sites, we applied a linear regression method to systematically quantify the influence of *cis*-elements in this region on inosine deposition. This approach has been validated as effective in computational analyses of m^6^A deposition^47,48^ (Fig. 6a). As saturated mutations at each nucleotide concurrently create and disrupt five overlapping pentamers, linear regression was performed on the aggregated data to determine the net effect of each pentamer motif based on slope and statistical significance (see Materials and Methods for details). To further characterize the *cis*-regulatory code of A-to-I editing, we ranked all 5-mer motifs according to their regression coefficients and classified motifs with positive coefficients as enhancers and those with negative coefficients as silencers. These classifications represent the relative contributions of individual motifs to the predicted editing probability and should not be interpreted as absolute determinants of editing levels. Pentamer motifs were ranked by their effect magnitude and statistical significance, with the top 10 enhancers and silencers displayed. The results revealed that inosine enhancers predominantly contain partial TAG motifs, whereas inosine silencers are characterized mainly by GG/AG dinucleotides (Fig. 6b, c). Positional distribution analysis within the –10 nt to +30 nt region revealed that the partial TAG enhancer motifs were strongly enriched at positions –2 and 0 relative to the editing site, indicating that these *cis*-elements function in close proximity to or directly overlapping the edited adenosine. In contrast, GG/AG silencer motifs exhibited a broader positional distribution, with enrichment extending from the editing site to approximately +20 nt downstream (Supplementary Fig. S21).

To dissect the interplay between these *cis*-elements, we analyzed the co-occurrence matrix for 7072 sites with exactly one enhancer (E) and one silencer (S). The joint editing rates revealed a context-dependent mode rather than a fixed hierarchy. For instance, TAGAA successfully buffered the widespread repression of the AGGAG silencer, restoring the rate from 0.391 to 0.50. Element stratification further demonstrated distinct dosage and competitive effects (Supplementary Fig. S22a). Pure enhancers showed a non-linear dosage effect peaking at E = 2 (Δ = +0.073), whereas pure silencers displayed progressive linear repression. Notably, in co-occurring contexts, enhancer-dominant sites (E > S) net a marked increase (Δ = +0.070), whereas silencer-dominant sites (S > E) showed only mild repression (Δ = –0.017), indicating that editing output is driven by a competitive balance between opposing forces (Supplementary Fig. S22b).

Next, we explored the influence of the distribution of enhancer and silencer motifs in the flanking regions of inosine sites on editing levels. To rule out opposing effects caused by synergistic or competitive interactions on RNA editing when multiple enhancers or silencers are present, we first excluded sequences containing multiple silencers or multiple enhancers and analyzed the impact of a single type of enhancer or silencer on RNA editing efficiency. The results demonstrated that editing levels were higher when inosine enhancers were located around inosine sites. For instance, inosine sites containing the strong-effect enhancer TTTAT exhibited significantly elevated editing levels (Fig. 6d). Conversely, inosine sites with inosine silencers, such as the silencers GGGAG, CGGAG, and AGGAG, showed significantly lower editing rates. Statistical analysis of editing rates at inosine sites adjacent to the top 10 enhancers and silencers within the –10 nt to +30 nt region revealed that enhancers tended to be distributed at high-editing-rate sites, whereas editing rates at inosine sites surrounding silencers were significantly lower than those near enhancers (Supplementary Fig. S23). These observations regarding the differential association between the distribution of enhancers/silencers and inosine site editing levels support the hypothesis that functional *cis*-elements are primarily localized to this region of inosine sites. These findings provide a crucial theoretical basis for understanding the precise regulatory mechanisms underlying inosine modification.

## Discussion

RNA editing regulates protein function by altering RNA sequences without modifying DNA, influencing splicing, translation, and RNA stability, and serves as a key regulatory layer that links genotype to phenotype^49^. Accurate identification of RNA editing sites is crucial for understanding biological processes, disease mechanisms, and development of novel therapies^50–53^. The presence of false positives or false negatives in RNA editing site detection may introduce systematic statistical biases into downstream disease association analyses, potentially yielding invalid biological interpretations^54^. Current RNA editing detection methods still face significant challenges, including confounding by SNPs, sequencing errors, and high false-positive rates in non-Alu regions. To date, RNA-seq methods for identifying RNA editing sites remain dominant in clinical research^55–57^, primarily due to their operational convenience and the dual advantage of enabling the simultaneous detection of inosine modification levels and expression analysis. However, this approach has technical limitations in precisely identifying inosine sites, as false-positive sites cannot be completely eliminated. Researchers must exercise caution when investigating inosine-disease links, as some cases require resource-intensive DNA sequencing for validation. Particularly in studies on RNA viruses, given their high mutation propensity and lack of DNA genomic information, distinguishing between mutation events and ADAR-mediated editing sites becomes extremely challenging^58^. The increasing therapeutic potential of RNA editing demands highly sensitive and accurate detection methods. Developing a universal quantitative detection platform would significantly advance RNA editing research, enabling a deeper understanding of its biological roles, disease mechanisms, and therapeutic applications.

To address the limitations of current RNA editing detection methods, we developed REX-seq, an efficient two-step enzymatic method for precise RNA editing identification. The innovation of this technology lies in its integrated design, which combines RNA editing detection with expression analysis within a single experimental workflow. Compared with other RNA editing detection methods (e.g., EndoVIPER-seq, hEndoV-seq, and Slic-seq enrichment protocols), REX-seq employs an optimized two-step enzymatic treatment that not only enables accurate quantification of RNA editing sites but also preserves the original transcript expression profiles. This “dual-dimensional detection” capability enables researchers to simultaneously obtain correlated data on editing dynamics and expression patterns in a single experiment, effectively overcoming the quantitative bias and loss of expression information caused by enrichment steps in conventional methods. Notably, inter-replicate variability in editing sites was observed (Supplementary Fig. S3a). This variability may reflect the dynamic nature of the RNA secondary structure, which can influence ADAR accessibility and thereby contribute to differences in editing sites between replicates rather than solely representing technical variability.

Furthermore, the antibody- and compound-free design of REX-seq streamlines experimental procedures, lowers technical barriers, and enhances its applicability in both clinical and basic research. With wide applicability in various biological samples, REX-seq offers a powerful platform for studying the biological roles of RNA editing and its regulatory relationships with gene expression. Our REX-seq analysis revealed 84,645 new editing sites predominantly located in low-efficiency regions, indicating significant 5′UTR and CDS enrichment. These sites, potentially undetected by conventional methods, may have functional significance. For instance, *RBFA* and *NECAB3* editing sites could modulate pre-mRNA splicing or protein sequences, implicating them in neurodevelopmental and immune regulatory pathways. However, the XRN-1 digestion step, although essential for accurate quantification, introduces a mild 5′ bias in clustered editing regions. Systematic evaluation against conventional RNA-seq confirmed that this bias is confined to the first ~50 bp of clusters and is quantitatively limited (Δ < 0.1). Importantly, editing levels at sites beyond 50 bp are reliably recovered and show high concordance with RNA-seq measurements. This trade-off should be considered when interpreting editing dynamics in Alu-rich regions. In addition, while independent experimental replication confirmed the detectability of a substantial fraction of newly identified sites, we acknowledge that site-specific orthogonal validation (e.g., targeted amplicon sequencing or RT-PCR) remains the gold standard for authenticating low-abundance editing events. Future studies employing such approaches will be valuable to further substantiate the biological relevance of these novel sites.

While the initial development of InoDREAM capitalized on the deep sequencing coverage of HEK293T cells, extending our evaluation to broader datasets is essential to verify the generalizability of the model. To address the limitation of relying solely on a single cell line, the comparative evaluation between the HEK293T and REDIportal-trained models provides crucial insights into the generalizability of InoDREAM. The minor performance trade-off observed is biologically expected, as the HEK293T model benefits from a highly homogeneous isogenic distribution, whereas the REDIportal model must navigate substantially greater sequence and tissue diversity. Crucially, the robust performance of the REDIportal-trained model across diverse cell types, in addition to its high transferability to independent REX-seq data, confirms that InoDREAM successfully captures broad, evolutionarily conserved sequence features of cellular A-to-I editing rather than cell line-specific artifacts. This comparison substantially reinforces the robustness of our framework for broader pan-tissue applications.

Despite the identification of millions of inosine sites via high-throughput sequencing technologies in recent years, our knowledge regarding the potential biological functions of inosine modifications remains relatively limited. This field is still confronted with numerous scientific questions awaiting resolution. First, the regulatory protein network that specifically interacts with RNA editing sites has not been systematically characterized. Second, whether these sites are subject to precise regulation by specific *cis*-elements (e.g., editing-dependent enhancers or silencers) remains elusive. Third, research into the mechanism underlying the association between dynamic changes in inosine modifications and gene expression regulatory networks is also significantly delayed. The persistent existence of these fundamental issues has severely impeded our in-depth understanding of the biological functions of inosine modifications and has constrained the translation of relevant research findings into clinical applications. Particularly in disease-related studies, owing to the lack of a comprehensive understanding of the regulatory mechanisms governing inosine sites, researchers frequently encounter difficulties in accurately differentiating pathogenic editing events from nonsense variations, which has emerged as one of the most prominent challenges in the current field of inosine research.

Following the successful identification of high-quality RNA editing sites, we further focused on dissecting the regulatory mechanisms underlying these editing events. We innovatively integrated high-precision REX-seq data with deep learning techniques to develop the InoDREAM model. Through interpretability analysis of the InoDREAM model, we identified multiple RBP-binding motifs associated with neurogenesis and RNA splicing (such as *MSI1*, *PTBP1*, and *SRSF7*), suggesting that RNA editing may form a cooperative regulatory network with specific RBPs by dynamically altering local sequence features. A key conceptual consideration in interpreting our integration of RNA editing profiles and RBP binding motifs is the directionality of causality. While enriched motifs suggest potential functional cross-talk, it remains biologically plausible that the relationship is bidirectional: RNA editing may alter RBP binding affinity, or conversely, resident RBPs may modulate ADAR recruitment by acting as structural enhancers or silencers. Although our preliminary siRNA knockdown experiments targeting MSI1 and PTBP1 yielded minor global effects on editing levels, the regulatory interplay between the analytical code of RBPs and ADAR accessibility is likely highly context-dependent and characterized by functional redundancy. Therefore, future high-throughput systematic perturbation studies encompassing a broader spectrum of RBPs and experimental conditions will be essential to fully decouple these bidirectional mechanisms and definitively map the causal networks governing A-to-I editing regulation.

The distinct –1 nucleotide preference and unique enrichment of novel sites within the 5′ UTR and CDS regions may reflect the intrinsic selectivity of ADAR enzymes under non-canonical structural and sequence contexts. Methodologically, unlike conventional algorithms that rely on high-density Alu clusters, our dual-signal framework captures scattered, low-abundance sites that are often overlooked. Although enzymatic digestion during library preparation could theoretically introduce minor cleavage biases, our downstream bioinformatic filtering, which stringently factors in mutation positions and ratios, minimizes potential experimental artifacts, suggesting that these features represent genuine biological traits.

The InoDREAM model not only achieves high-precision prediction of A-to-I editing sites (AUROC = 0.965) but also reveals the critical role of *cis*-elements in specific regions. This study revealed that the *cis*-elements regulating inosine deposition are distributed mainly in the –10 nt to +30 nt region, among which specific pentamer motifs (such as TTTAT and GGGAG) act as enhancers and silencers, respectively, significantly affecting editing efficiency. However, a limitation of this study is that the identification of the *cis*-regulatory elements and motifs by InoDREAM is entirely based on computational predictions and lacks direct experimental validation. Future work will incorporate experimental approaches, such as eCLIP, RNA immunoprecipitation, and targeted editing assays, to physically validate these predictions and establish definitive causal regulatory mechanisms. In addition, the model successfully predicts the impact of pathogenic SNVs on inosine deposition (e.g., mutations in the *FLNA* and *TTC7A* genes), providing a new annotation dimension for research on disease mechanisms. Notably, the unique value of InoDREAM lies in its ability to independently evaluate regulatory region variants that do not alter protein sequences, supplementing the existing disease mutation annotation system centered on coding regions and providing an innovative tool for RNA epigenetic research in the era of precision medicine. Importantly, our in silico analysis highlighted a 40-mer core motif (–10 to +30) as a key *cis*-regulatory determinant of editing efficiency. While this computational prediction awaits direct experimental validation, it holds substantial relevance for the expanding field of site-directed RNA editing. This 40-mer region could offer a structural blueprint for designing programmable guide RNAs (gRNAs) or engineered antisense oligonucleotides. Integrating these specific *cis*-element features into therapeutic gRNA scaffolds could facilitate optimal ADAR recruitment and enhance on-target editing efficiency. In addition, although we systematically identified SNV-editing associations computationally, validation using population-scale datasets such as GTEx was not performed in this study. Incorporating such genomic resources to explore allele-specific effects represents a valuable direction for future research.

Although the REX-seq method and the InoDREAM interpretable neural network model have demonstrated excellent performance in RNA editing research, this study still has certain limitations. The primary limitation is that the existing data analysis relies mainly on sample information from the HEK293T cell line and lacks differential analysis of RNA editing in clinical disease cohorts, which to some extent restricts the clinical translational value of the research findings. Although the training data of InoDREAM cover a large number of high-confidence sites, it has not yet integrated editing data from more cell types or pathological states. Future studies could further optimize the technical workflow, expand the application scope of the model, and combine functional experiments to verify the biological effects of newly discovered sites. The 201-bp input window in InoDREAM captures local features around editing sites but does not model long-range dsRNA structures, which ADAR enzymes can recognize across hundreds or thousands of nucleotides. Although the local sequence context provides substantial predictive power, incorporating broader structural information may further improve performance. Future work integrating global RNA folding could address this limitation. In addition, exploring the synergistic effects between RNA editing and other epitranscriptomic modifications (such as m^6^A) will help elucidate the complexity of post-transcriptional regulatory networks^59^. In summary, by integrating dual-enzyme activity for RNA editing detection with a deep learning model, this study provides high-precision tools for identifying RNA editing sites and offers new mechanistic perspectives on both the molecular regulation of RNA editing and its pathophysiological roles in disease progression.

## Materials and Methods

### Chemicals and reagents

Single-stranded oligonucleotides with or without inosine (I-RNA and A-RNA) were synthesized by Takara Bio, Inc. The detailed sequences are listed in Supplementary Table S1.

### Cell culture

HEK293T cells were cultured at 37 °C under 5% CO_2_ in DMEM (Life Technologies) supplemented with 10% FBS and 1% penicillin/streptomycin (Gibco, Beijing, China).

### Procedure of poly(A) + RNA isolation and fragmentation

Total RNA was isolated from cell lines with TRIzol (Invitrogen) according to the manufacturer’s protocol. The mRNA was isolated by subjecting total RNA to oligo(dT) enrichment using Dynabeads Oligo(dT)_25_ (NEB), and the gDNA was removed by DNase I (Vazyme). Purified polyadenylated RNA was fragmented using RNA fragmentation reagents (NEB) at 94 °C for 4 min. Human brain poly(A) + RNA was purchased from Takara Bio, Inc.

### Validation of the effects of enzymes on RNA using oligonucleotides

To assess the enzyme activity of EndoV (NEB) and XRN-1 (NEB), a 60-mer inosine-containing single-stranded RNA (I-RNA; Supplementary Table S1) and its control counterpart lacking inosine (A-RNA; Supplementary Table S1) were employed as substrates. The enzymatic reaction was performed in a 10 μL system containing 100 ng of I-RNA/A-RNA with a standard amount of EndoV. The reaction mixture was incubated at 37 °C for 60 min. Following purification, the reaction products were subsequently treated with XRN-1 at 37 °C for 60 min. The reactions were terminated by adding 1 μL of 10× loading buffer (Takara). Cleaved products were analyzed by 20% SDS-PAGE. The polyacrylamide gel was stained with GelRed (Biosharp) and visualized using an imager ChemiDoc XRS+ Imaging System (Bio-Rad).

### Verification experiment of XRN-1-specific digestion

To verify XRN-1-specific digestion, 2 μg of mRNA extracted from 293T cells was fragmented at 94 °C for 4 min via ion fragmentation, generating RNA fragments with 5′-OH and 3′-Pi termini. The purified fragmented RNA was equally divided into four groups for different treatments: the negative control group without enzymatic treatment, the XRN-1 single-enzyme group incubated at 37 °C for 30 min, the T4 PNK single-enzyme group reacted at 37 °C for 30 min, and the dual-enzyme group sequentially treated with T4 PNK followed by XRN-1. All the samples were subsequently purified after the reactions and were subjected to 2% agarose gel electrophoresis with imaging analysis.

### Construction of a knockout cell line by CRISPR-Cas9

CRISPR gRNA targeting the *ADAR1* gene was annealed into double-stranded oligonucleotides and ligated into the PX459 plasmid purchased from Addgene. The recombinant PX459-gRNA plasmids were directly transfected into target cells.

Post-transfection cells were collected and seeded into 96-well plates via limiting dilution at a density of one cell per well to isolate single-cell clones. After two weeks of cultivation, puromycin selection was performed to obtain stably edited single clones, followed by large-scale expansion of resistant cell clones. Candidate knockout clones were first screened by Western blot to detect the absence of the ADAR1 protein. Genomic DNA was extracted from positive clones, and the target gene fragment was amplified by PCR, followed by Sanger sequencing to confirm successful gene disruption at the genomic locus.

### REX-seq library preparation

A library was prepared following a previously reported procedure with slight changes^17^. Fragmented RNA was first subjected to 3′-end repair by T4 PNK (NEB). First, the RNA was treated with EndoV for cleavage. Afterward, XRN-1 was used to digest the cleaved fragments. 3N-adapter ligation (/5rApp/NNNTGGAATTCTCGGGTGCCAAGG/3ddC/) followed the digestion, and the 3′ adapter was removed by 5′-deadenylase (NEB) and RecJf (NEB) digestion. After purification, SuperScript IV (Thermo Scientific) was used to perform reverse transcription (primer, 5′-ACACGACGCTCTTCCGATCT-3′). After cDNA synthesis and purification, 5′ adapter (5′-/Phos/NNNNNNNNNNAGATCGGAAGAGCACACGTCTG/3ddC/) ligation was performed. After purification with MyOne silane beads (Thermo Scientific), qPCR was performed to evaluate the cycle numbers of each sample to avoid over-amplification. Library PCR amplification was performed using NEB primers. The library products were subsequently purified by Vazyme Purification Beads or low-melting point agarose gel electrophoresis, after which the Illumina NovaSeq 6000 platform with a PE150 read length was used for sequencing (LC-Bio Technology, China).

### Sequencing data processing

The 150 bp paired-end reads from the Illumina NovaSeq 6000 system sequencing were first sent for adapter and quality trimming using trim galore, and reads shorter than 30 nt after trimming were excluded. Note that we included a random decamer (NNNNNNNNNN) at the 3′ end and a random trimer (NNN) at the 5′ end, ligated to the fragments during library construction. Terminal barcodes prevented read-end sequencing errors, ensuring full-length read accuracy. Random barcodes were used to distinguish PCR duplicates from unique fragments with identical sequences. PCR duplicates were removed using Fastuniq. After the random barcodes were trimmed, the clean reads were first mapped to ribosomal RNAs, and the unmapped reads were subsequently aligned to the hg38 genome using HISAT2 with the parameters ‘--mp 4,2 --rna-strandness RF --no-softclip’. Only properly paired primary alignments were retained for downstream analysis.

### Identification of putative inosine sites

Mutation profiles were analyzed using a custom script that converted the pileup format into a tabular summary of mutations at each genomic position. Separate analyses were performed for positive- and negative-strand genes. Truncated read analysis was based on the specific biochemical properties of EndoV, which cleaves precisely at the first phosphodiester bond 3′ of inosine (1 bp downstream). During reverse transcription, the reverse transcriptase terminates at this physical nick, generating a 3′-end truncation signal in the sequencing read. Simultaneously, inosine pairs with guanine during reverse transcription, producing an A-to-G mismatch at the penultimate position of the truncated read. Therefore, a genuine EndoV cleavage event exhibits two spatially coupled features: a truncation end and an A-to-G mismatch at the immediately preceding nucleotide. For the truncated read analysis, only mismatch events meeting two requirements were considered, which included at least six mismatches and a minimum of 10% truncation read coverage. Adjacent A-to-I editing sites potentially lost during cleavage were identified using relaxed criteria, which required at least four mismatches with 5% truncation coverage within 3 bp of strictly identified sites. Final A-to-I editing candidates were defined as A-to-G conversions in the positive strand or T-to-C conversions in the negative strand with Phred quality scores of at least 27 at the second read position after all the filtration steps. Because REX-seq libraries are strand-specific, editing events were initially identified independently on forward and reverse reads. To unify the statistical analysis, mismatches detected on the negative strand were reverse-complemented to their positive-strand equivalents (e.g., T → C on the negative strand was converted to A → G). These converted mismatches were then merged with mismatches from the positive strand for downstream analysis, ensuring that all editing events were represented in a single, strand-unified framework. This processing step was applied prior to all subsequent analyses, including editing frequency calculation, replicate overlap assessment, and motif enrichment.

### Annotation of inosine sites

The editing sites were annotated using ANNOVAR^60^ to determine their genomic locations within host genes. Gene Ontology enrichment analysis was performed using DAVID online bioinformatics tools, and the results were analyzed by Fisher’s exact test (https://david.ncifcrf.gov/).

### Overview of InoDREAM

In this study, we employed two distinct DNN architectures — CNNs and recurrent neural networks (RNNs) — to effectively learn sequence features for RNA inosine site prediction. The model begins by processing input RNA sequences of 201 nt, and the input sequences are then encoded into binary vectors based on a one-hot encoding strategy, in which A, C, G, and T are encoded as [1,0,0,0], [0,1,0,0], [0,0,1,0], and [0,0,0,1], respectively. The feature extraction pipeline employs a biologically inspired design starting with a motif-scanning convolutional layer utilizing 256 filters with 7-nt receptive fields, batch normalization (BatchNorm) layers, and rectified linear units (ReLU). The outputs of the first layer are preserved for subsequent motif analysis. The architecture then processes these primary motif features through a multi-scale residual block containing parallel convolutional pathways with 3-nt and 11-nt kernels operating simultaneously, enabling the model to capture both local sequence contexts and broader structural patterns while maintaining feature dimensions through residual connections. Afterward, features are processed through 256-dimensional positional encoding followed by a bidirectional LSTM layer (128 hidden units per direction) to capture sequential relationships. The position encoding is calculated by

1 $$P{E}_{\left({pos},2i\right)}=\,sin \left(\frac{{pos}}{{10000}^{2i/{d}_{{emb}}}}\right)$$

2 $$P{E}_{\left({pos},2i+1\right)}=cos \left(\frac{{pos}}{{10000}^{2i/{d}_{{emb}}}}\right)$$

where pos is the position of the token in the sequence and i is the index of dimension, with the embedding dimension d_emb_ = 256. In the Transformer’s self-attention mechanism, the input sequence embedding X is multiplied by three learnable weight vectors, W_q_, W_k_, and W_v,_ to be converted to the query, key, and value vectors (Q, K, V). The scaled dot-product attention is then computed as

3 $${Attention}\left(Q,K,V\right)={softmax}\left(\frac{Q{K}^{T}}{\sqrt{{d}_{k}}}\right)V$$

where d_k_ is the dimension of Q and K. Then, an 8-head attention mechanism is applied to identify and weight important sequence positions. These attention-weighted features are fed into a final classifier comprising a 512-unit activated dense layer and a 2-unit output layer. The implementation employs 20% dropout regularization and AdamW optimization. For interpretability, the architecture supports convolutional filter visualization for motif identification, attention weight analysis to pinpoint significant sequence regions, and multi-scale feature examination across spatial resolutions, providing both predictive accuracy and biological insights into RNA modification patterns.

### Datasets

The dataset for the InoDREAM deep learning framework was derived from a high-confidence A-to-I editing site dataset generated using our REX-seq method. During data processing, we extracted genomic sequences centered on each inosine site, spanning 100 bp upstream and downstream (total length: 201 bp) as the feature region. To eliminate sequence redundancy, we applied stringent filtering using the CD-HIT^61^ program with a sequence similarity threshold of 80%, which is a widely accepted standard in genomic sequence analysis. Through this quality control pipeline, we obtained 186,028 non-redundant, high-confidence editing site sequences, providing a robust training foundation for neural network modeling. To construct highly reliable negative controls, we implemented a dual-selection strategy: (1) strict genomic matching by randomly selecting an equal number of unmodified sites from the human genome, along with their adjacent upstream and downstream regions, and (2) non-overlapping criteria where all negative sites were rigorously verified to avoid any genomic coordinate overlap with positive sample regions. For each selected negative site, we similarly extracted 100 nt of the flanking sequence (total length: 201 nt), maintaining identical feature dimensions to those of the positive samples. The final dataset was randomly partitioned into training, testing, and validation sets at a 6:2:2 ratio. This partitioning strategy ensures balanced data distribution while effectively assessing model generalizability, with detailed sample sizes and dataset characteristics provided in Supplementary Table S2.

### Model training and testing

Model training and optimization were implemented using PyTorch with CUDA acceleration, with all random seeds fixed to ensure reproducibility. Network weights were initialized using Xavier uniform initialization for both linear and convolutional layers. We employed the Adam optimizer (β₁ = 0.9, β₂ = 0.999, and ε = 1e–8) with L2 weight decay (λ = 1e–4) and learning rates between 1e–4 and 1e–3, using mini-batch gradient descent with a batch size of 128 across 100 training epochs. The training process incorporated a “save best” model checkpoint system that preserved weights only when the test accuracy improved, along with comprehensive performance monitoring tracking. The evaluation protocol calculated training loss via cross-entropy (nn.CrossEntropyLoss) and performed batch-wise gradient computation and parameter updates and automatic saving of the best-performing models. Hyperparameter optimization was conducted using the validation set, whereas the final model performance was assessed on the test set. All model computations were implemented on Nvidia Tesla A100 GPU computing platforms.

### Evaluation metrics

To evaluate the performance of InoDREAM, we employed the widely adopted assessment metrics: ACC, sensitivity (Se), specificity (Sp), MCC, and AUC, which can be expressed as follows:

4 $${ACC}\,=\frac{{TP}\,+\,{TN}}{{TP}\,+\,{FN}\,+\,{TN}\,+\,{FP}}$$

5 $${Se}\,=\frac{{TP}}{{TP}+{FN}}$$

6 $${Sp}\,=\frac{{TN}}{{TN}\,+\,{FP}}$$

7 $${MCC}=\frac{\left({TP}\,\times \,{TN}\right)-\,\left({FP}\,\times \,{FN}\right)}{\sqrt{\left({TP}\,+\,{FP}\right)\left({TP}\,+\,{FN}\right)\left({TN}\,+\,{FP}\right)\left({TN}\,+\,{FN}\right)}}$$

where TP represents true positive samples, TN represents true negative samples, FP represents false-positive samples, and FN represents false-negative samples. The MCC values range from –1 to 1, where a coefficient of +1 indicates a perfect prediction and –1 indicates total disagreement between the prediction and the observation. AUC is defined as the area under the ROC curve and was compared with reported performance metrics from related studies to assess relative predictive performance. Using the above evaluation metrics, we compared the modeling performance of InoDREAM with that of the deep learning-based EditPredict method^25^. Both models were trained on identical REX-seq-validated high-quality sites to ensure comparability.

### Position-specific mutational effects on inosine deposition profiled by single-nucleotide saturation mutagenesis

To identify sequence determinants of inosine deposition, for each inosine site, we performed systematic nucleotide substitutions (positions –100 to +100) with all three alternative bases and calculated the changes in the inosine probability value (ΔProbability) after mutation by InoDREAM. To ensure the reliability of ΔProbability measurements, we implemented stringent quality controls by exclusively analyzing inosine sites with baseline deposition probabilities ≥ 0.4, as predicted by InoDREAM. To quantify the effect of all the *cis*-element pentamers by linear regression, we followed the statistical approach used in a previously reported study^42^. Each nucleotide substitution simultaneously affected five overlapping 5-mer motifs, with disrupted motifs assigned –1 and newly created motifs assigned +1. Linear regression was performed for each 5-mer across all the data, with motif effects ranked by regression slope and statistical significance determined by the *P* value (Fig. 6a). This method allowed us to systematically evaluate the contribution of each 5-mer motif to inosine deposition probability. We subsequently evaluated the editing levels of inosine sites containing either top enhancer or silencer motifs. Specifically, we excluded inosine sites where multiple *cis*-elements (multiple enhancers, multiple silencers, or both enhancer and silencer motifs) were simultaneously present. This stringent filtering ensured that each analyzed site contained only a single significant *cis*-element. Finally, the top 10 enhancers and top 10 silencers were calculated. Finally, the top 10 enhancer and top 10 silencer motifs were identified based on their regression slope and statistical significance (*P* < 0.05), representing the most potent regulatory elements modulating inosine deposition.

### Characterization of SNVs linked to inosine modifications

We obtained variant data from the ClinVar database (https://ftp.ncbi.nlm.nih.gov/pub/clinvar/tab_delimited/variant_summary.txt.gz) and extracted SNVs located within ±100 nt of identified inosine sites. This approach allowed us to examine potential functional relationships between known genetic variants and inosine deposition positions. We classified the variants into three clinically relevant categories: (1) VUS, (2) benign/likely benign variants, and (3) pathogenic/likely pathogenic variants, based on their clinical annotations in ClinVar (Fig. 5g). To visualize the effect of SNVs on inosine deposition, saturation mutagenesis was performed in the region from –100 nt to +100 nt up- and downstream of inosine sites. Through a comprehensive analysis of ClinVar variants within inosine site flanking regions, we identified 213 mutations potentially associated with inosine deposition, and the ΔProbability of each mutation event is displayed as a heatmap (Fig. 5h).

## Supplementary information

Supplementary information

Supplementary Dataset S1

Supplementary Dataset S2

## Data Availability

The data that support this study are available from the corresponding authors upon reasonable request. The sequencing data generated in this study have been deposited into the Gene Expression Omnibus (GEO) under the accession number GSE305317. All other data needed to evaluate the conclusions in the paper are present in the paper and/or the Supporting Information. REXseq analysis pipelines, including a processing workflow and several Python scripts, were deposited on GitHub (https://github.com/sqhan-whu/rexseq). In addition, the deep learning framework and datasets of the inosine candidates are available on Zenodo (https://doi.org/10.5281/zenodo.16889881)^62^.
