# Supplementary material for: Deciphering the regulatory code of RNA inosine through enzymatic precision mapping and an explainable deep learning model
Source: Cell Discov. 2026 Nov 4;12:78. doi: 10.1038/s41421-026-00930-4 (PMC13737063; doi:10.1038/s41421-026-00930-4)
Supplement: Supplementary file 1 [file 41421_2026_930_MOESM1_ESM.pdf]

Deciphering the regulatory code of RNA inosine through enzymatic precision mapping and explainable deep learning model

Shaoqing Han[1*], Jiehua Li[1], Wei Yang[1], Qi Wei[1], Yafen Wang[3], Xiang Zhou[1,2*] and Xiaocheng Weng[1,2*]

[1]College of Chemistry and Molecular Sciences, Key Laboratory of Biomedical Polymers of Ministry of Education, Wuhan University, Wuhan, Hubei 430072, China

[2]Taikang Center for Life and Medical Sciences, Wuhan University, Wuhan, Hubei 430072, China

[3]School of Public Health, Wuhan University, Wuhan, Hubei 430071, China.

*Correspondence author (sqhan1@whu.edu.cn; xzhou@whu.edu.cn; xcweng@whu.edu.cn)

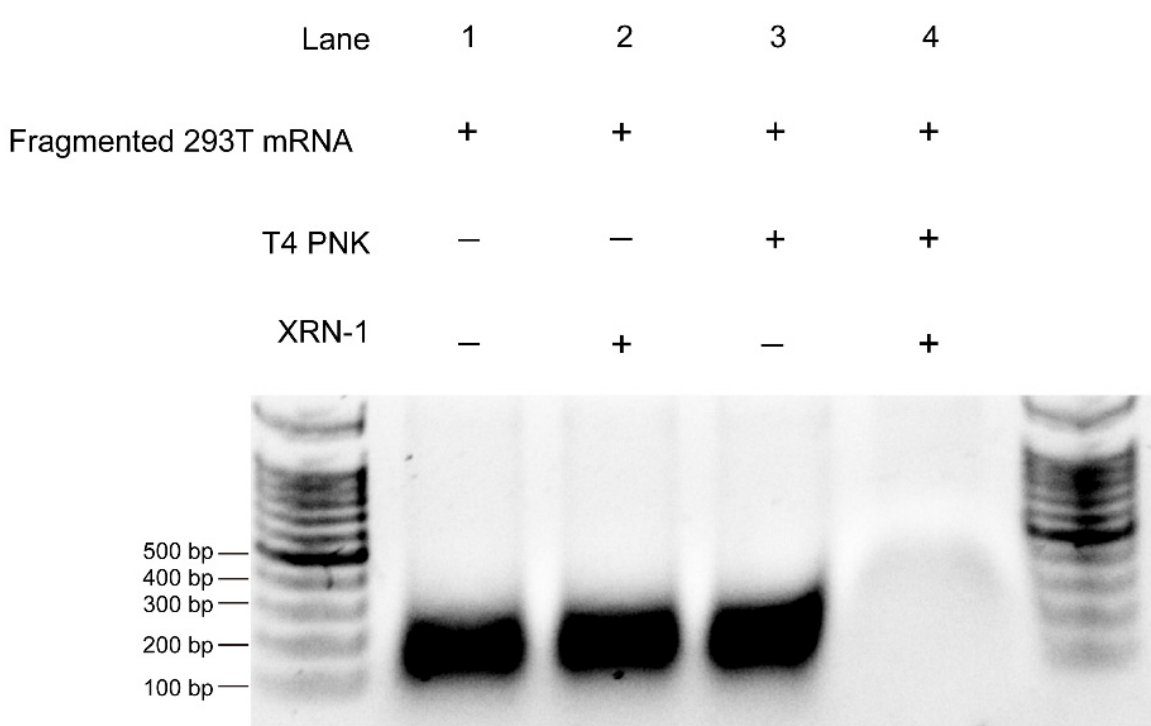

Supplementary Fig. S1 Agarose gel electrophoresis (2%) showing the digestion pattern of fragmented HEK293T mRNA upon treatment with T4 polynucleotide kinase (T4 PNK) and 5’→3’ exonuclease XRN-1. Lane 1: Fragmented mRNA only; Lane 2: Fragmented mRNA + XRN-1; Lane 3: Fragmented mRNA + T4 PNK; Lane 4: Fragmented mRNA + T4 PNK + XRN-1. XRN-1 only degrades RNA after T4 PNK-mediated 5′ phosphorylation, leading to complete RNA clearance in Lane 4. DNA ladder (100–500 bp) is shown on both sides as size reference.

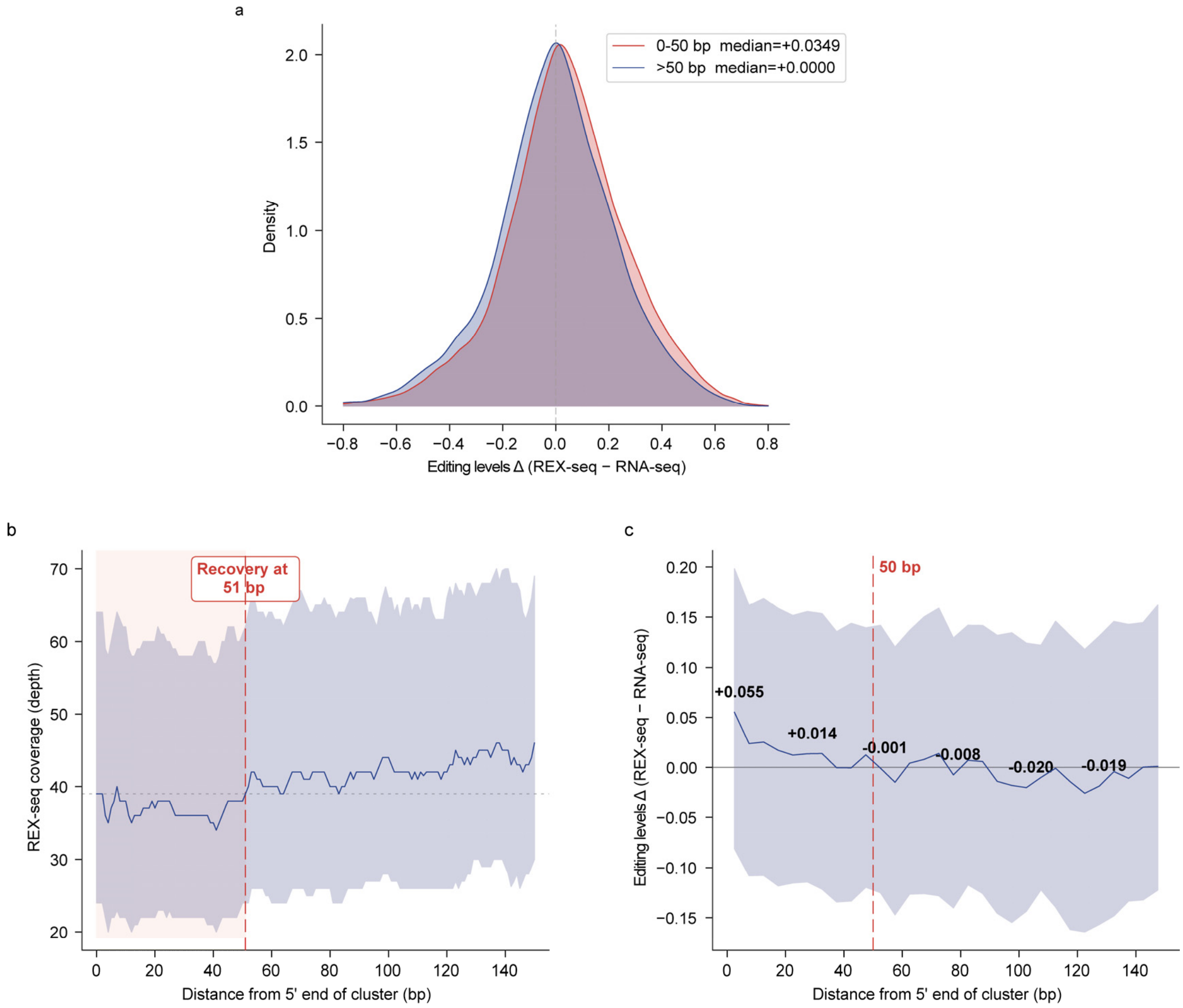

Supplementary Fig. S2 Combined analysis of REX-seq and RNA-seq editing levels at overlapping Alu sites. a Distribution of Δ (REX-seq editing level minus RNA-seq editing level) for sites within 0–50 bp (red) and >50 bp (blue) from the cluster 5′ end. Dashed line marks Δ = 0. **b** REX-seq coverage as a function of distance from the 5′ end of the cluster. Line shows median; shaded area shows interquartile range (IQR). **c** Mean Δ (REX-seq − RNA-seq) binned by distance from the 5′ end. Line shows mean; shaded area shows IQR.

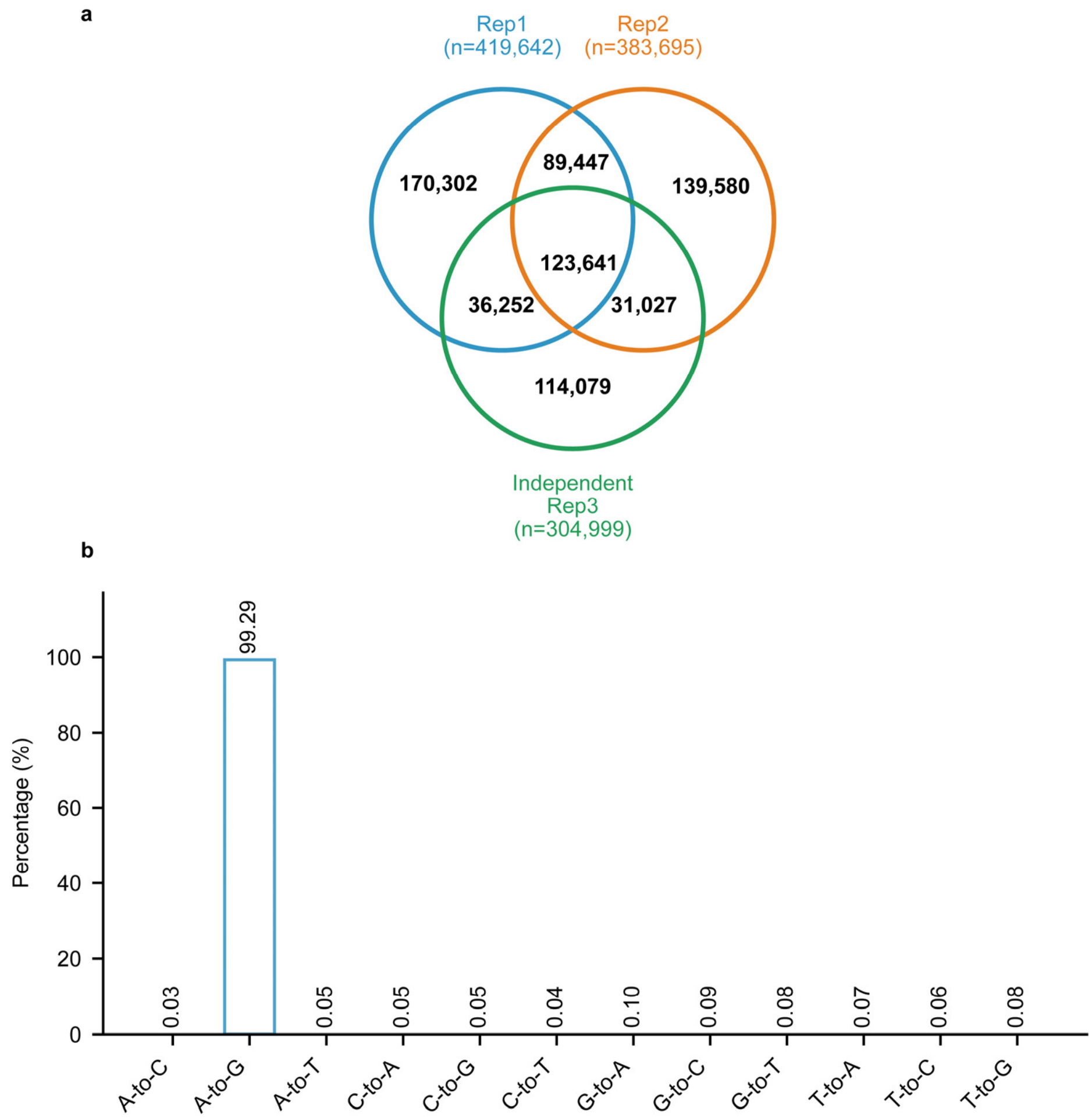

Supplementary Fig. S3 Independent validation of REX-seq reproducibility. a Venn diagram of editing sites from two original replicates and an independent experiment. The three-way intersection contained 123,641 sites. **b** Mutation type distribution of Rep3 sites. A-to-G substitutions accounted for 99.29% of all sites.

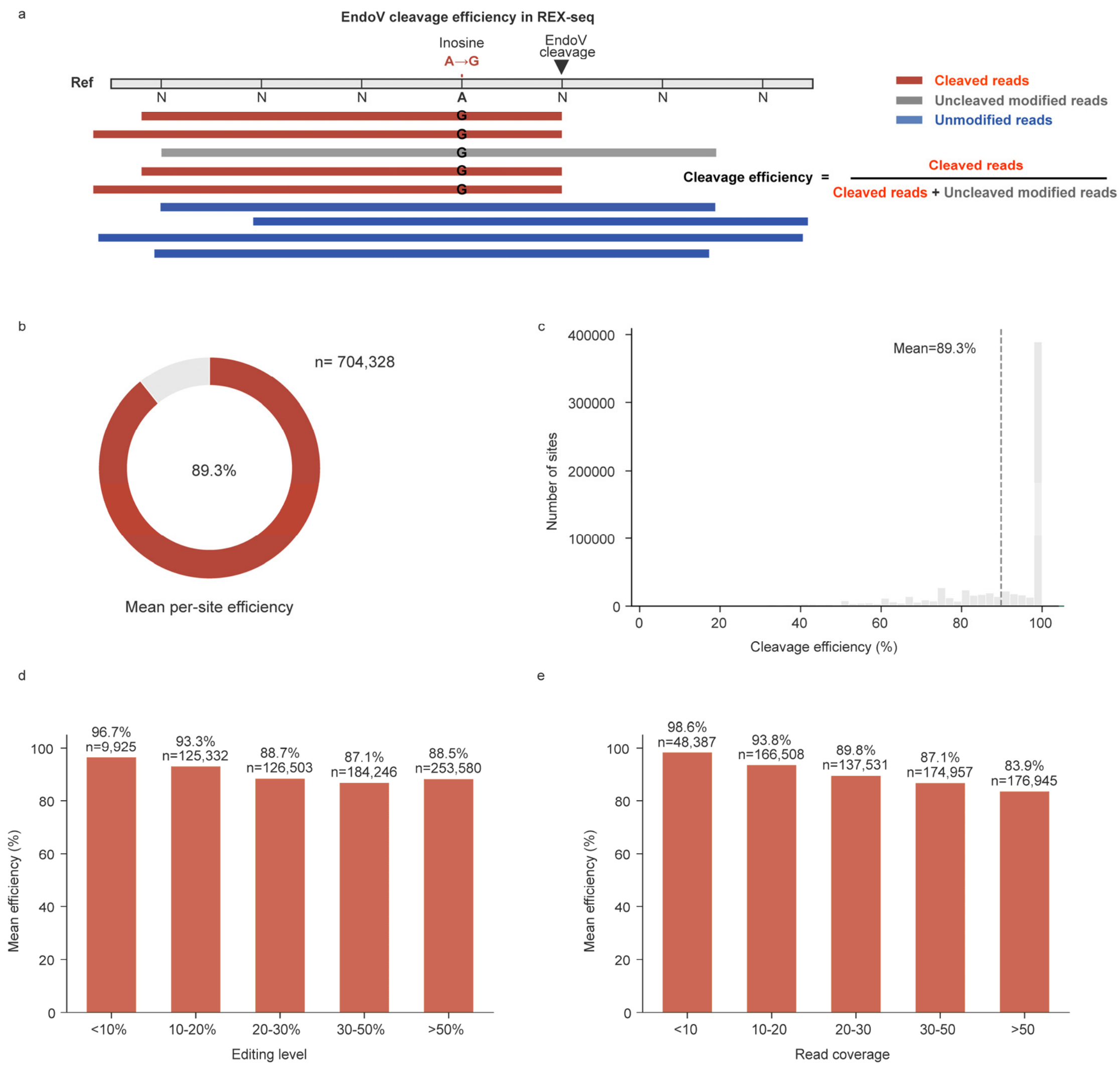

Supplementary Fig. S4 Evaluation of EndoV cleavage efficiency in REX-seq. a Schematic illustration of EndoV cleavage efficiency quantification. Inosine sites are cross-verified by both A-to-G mutations and truncated read analysis. Reads are categorized into three types: cleaved modified reads (orange), uncleaved modified reads (grey), and background unmodified reads (blue). Cleavage efficiency is calculated as the ratio of cleaved modified reads to the total number of modified reads (cleaved + uncleaved). **b** Global mean per-site cleavage efficiency. The donut chart illustrates the average cleavage efficiency across all detected inosine sites (n = 704,328). **c** Distribution of per-site cleavage efficiency. The histogram shows the number of inosine sites across different cleavage efficiency intervals, with a dashed line indicating the overall mean efficiency of 89.3%. **d** Mean cleavage efficiency across different RNA editing levels. **e** Mean cleavage efficiency across different sequencing read coverages.

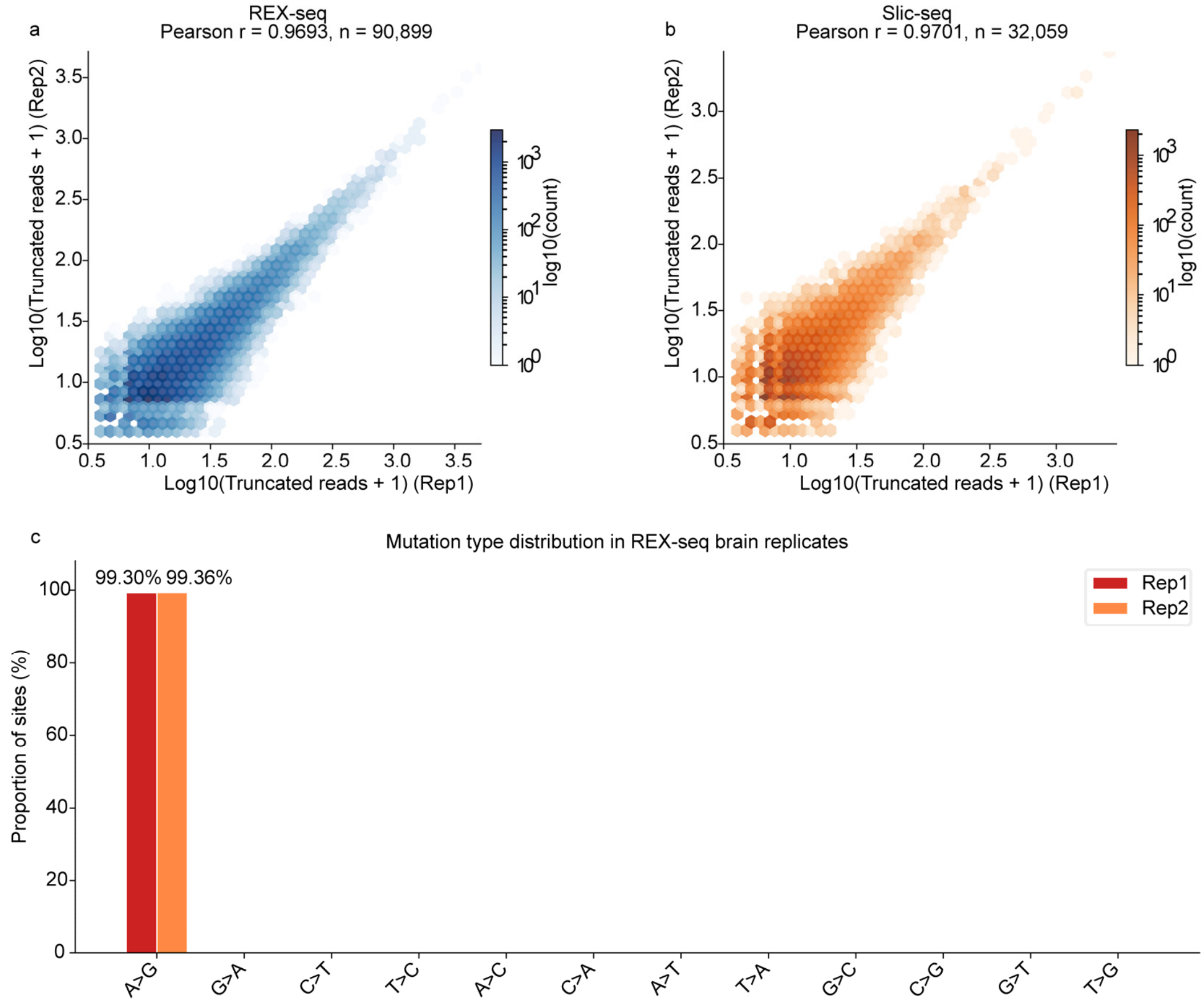

Supplementary Fig. S5 Reproducibility of REX-seq and Slic-seq detection and mutation type distribution. a, b Correlation analysis of $\log_{10}$-transformed truncated read counts between biological replicates of REX-seq (**a**) and Slic-seq (**b**). Pearson correlation coefficients and numbers of shared sites are indicated. **c** Mutation type distribution of editing sites detected in REX-seq brain replicates. The proportion of each of the 12 possible base substitution types is shown for both replicates.

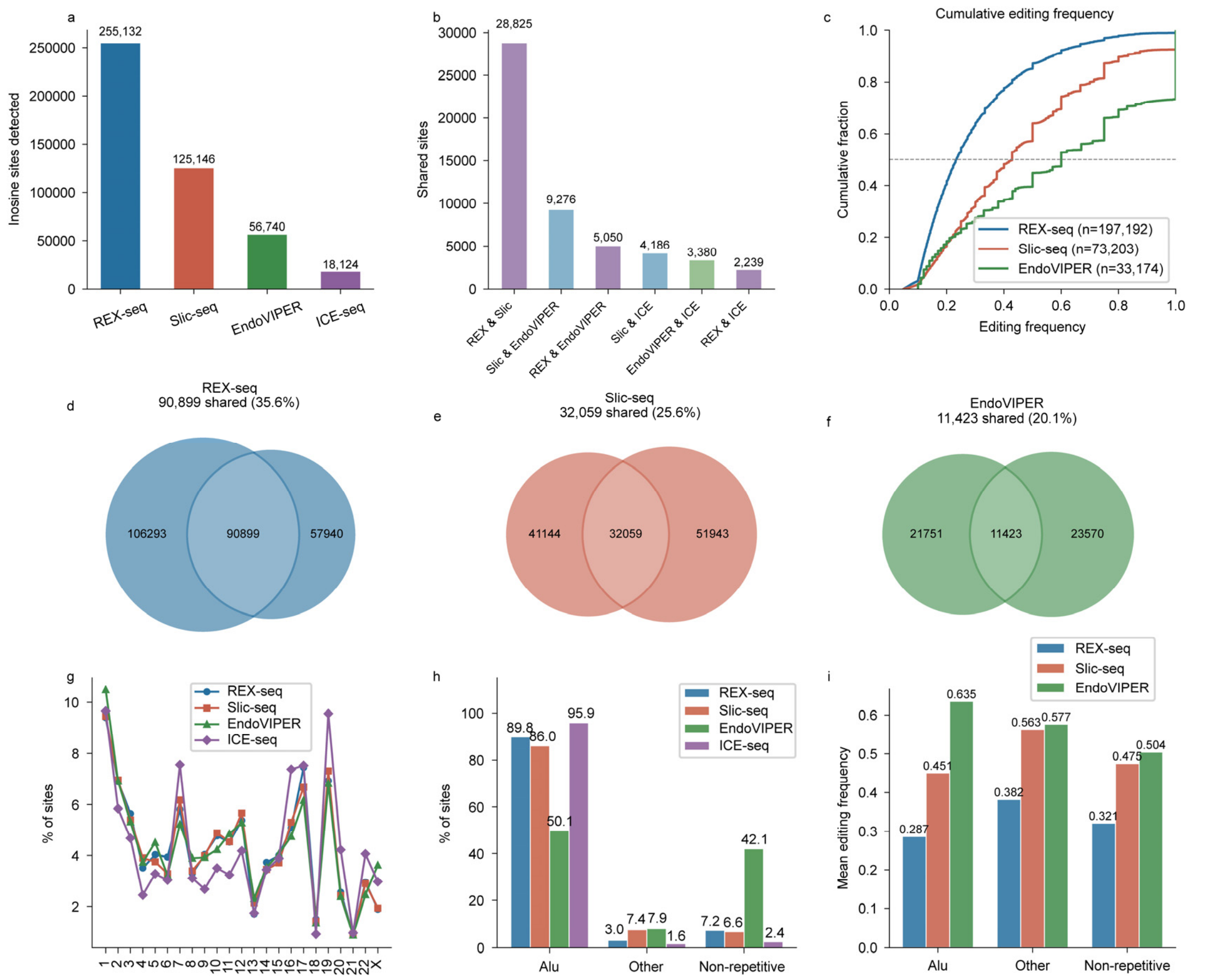

Supplementary Fig. S6 Comparison of REX-seq with published RNA editing detection methods. a Total number of unique editing sites detected by each method. REX-seq identified 255,212 sites, substantially more than Slic-seq (125,092), EndoVIPER (56,740), and ICE-seq (18,124). **b** Number of shared editing sites between method pairs. **c** Cumulative distribution of editing frequencies across all detected sites for each method. **d-f** Venn diagrams showing the overlap of editing sites between biological replicates for REX-seq (**d**), Slic-seq (**e**), and EndoVIPER (**f**). **g** Chromosomal distribution of editing sites. **h** Repeat element classification of editing sites. **i** Mean editing frequency across repeat element categories (Alu, Other, Non-repetitive) for each method.

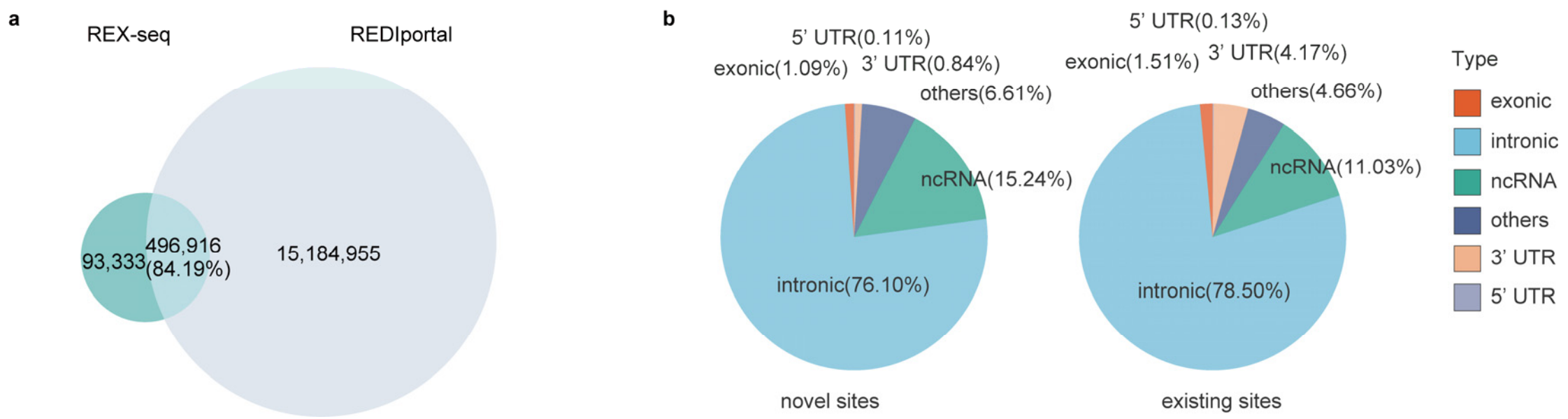

Supplementary Fig. S7 Characterization of inosine site detection and genomic distribution. a Venn diagram showing the overlap of inosine sites identified by REX-seq and REDIportal. The total number of sites in each dataset is indicated, with overlapping sites accounting for 84.19% of REX-seq-identified sites. **b** Pie charts comparing the genomic distribution of inosine sites between novel sites (identified in this study) and reported sites (from reported methods and public databases). Both datasets show predominant localization to intronic regions (76.10% for novel sites vs. 78.50% for existing sites), followed by non-coding RNA (ncRNA) regions (15.24% vs. 11.03%). Minor fractions are distributed across exonic regions, 3' UTRs, 5' UTRs, and other genomic elements, with percentages indicated for each category.

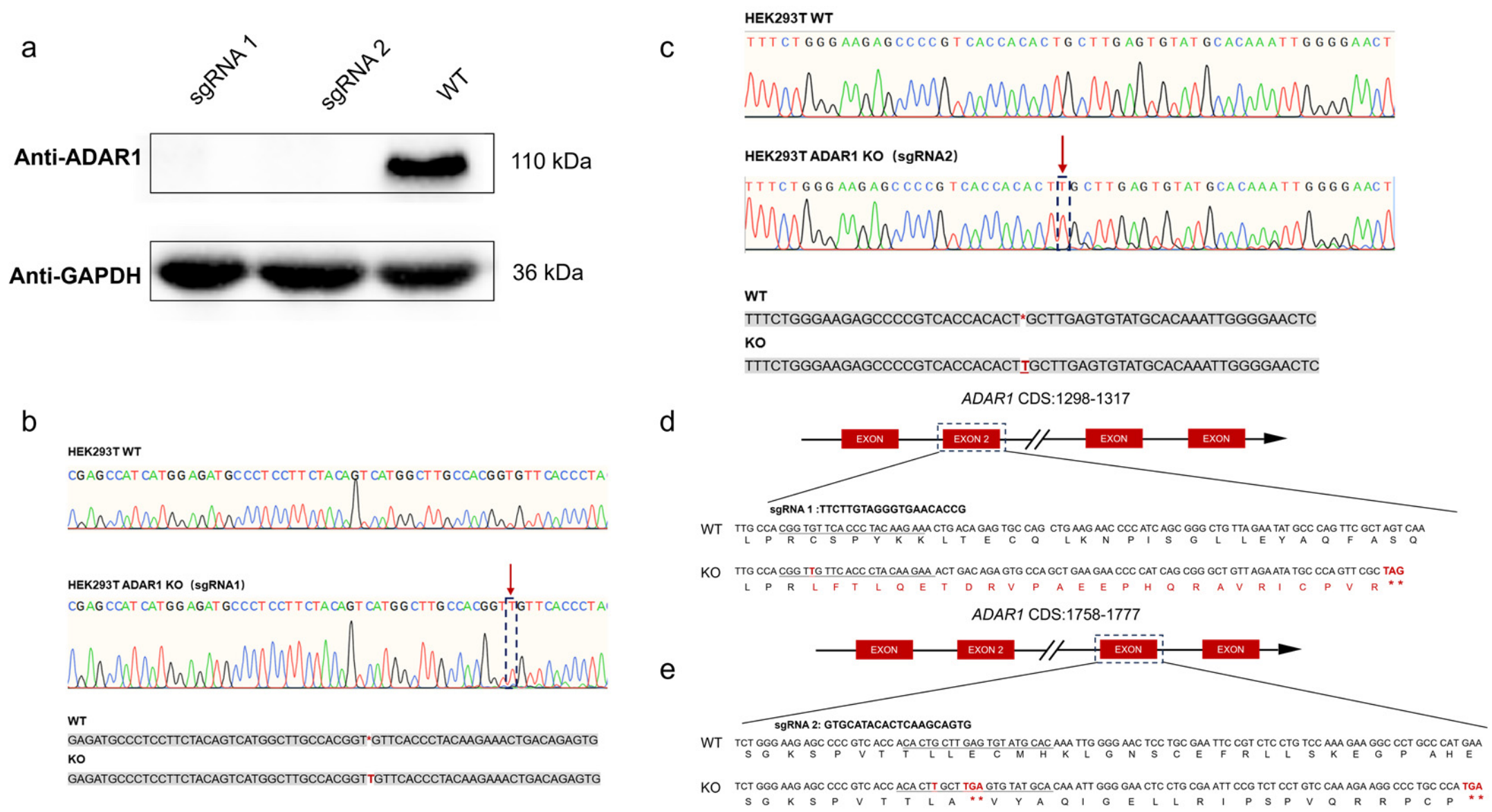

Supplementary Fig. S8 Generation of ADAR1 knockout cells. a Western blot validation of ADAR1 knockout efficiency. **b, c** Sanger sequencing validation of ADAR1 mutagenesis (**b** sgRNA1; **c** sgRNA2); wild-type and ADAR1 mutant sequences are shown. **d, e** Schematic illustration of ADAR1 knockout construction via the CRISPR-Cas9 system. The sgRNA sequence and mutant information are labelled. Single-nucleotide insertions were introduced at positions CDS1308 (**d**) and CDS1731(**e**) within the coding sequence, leading to premature stop codons at the corresponding amino acid positions.

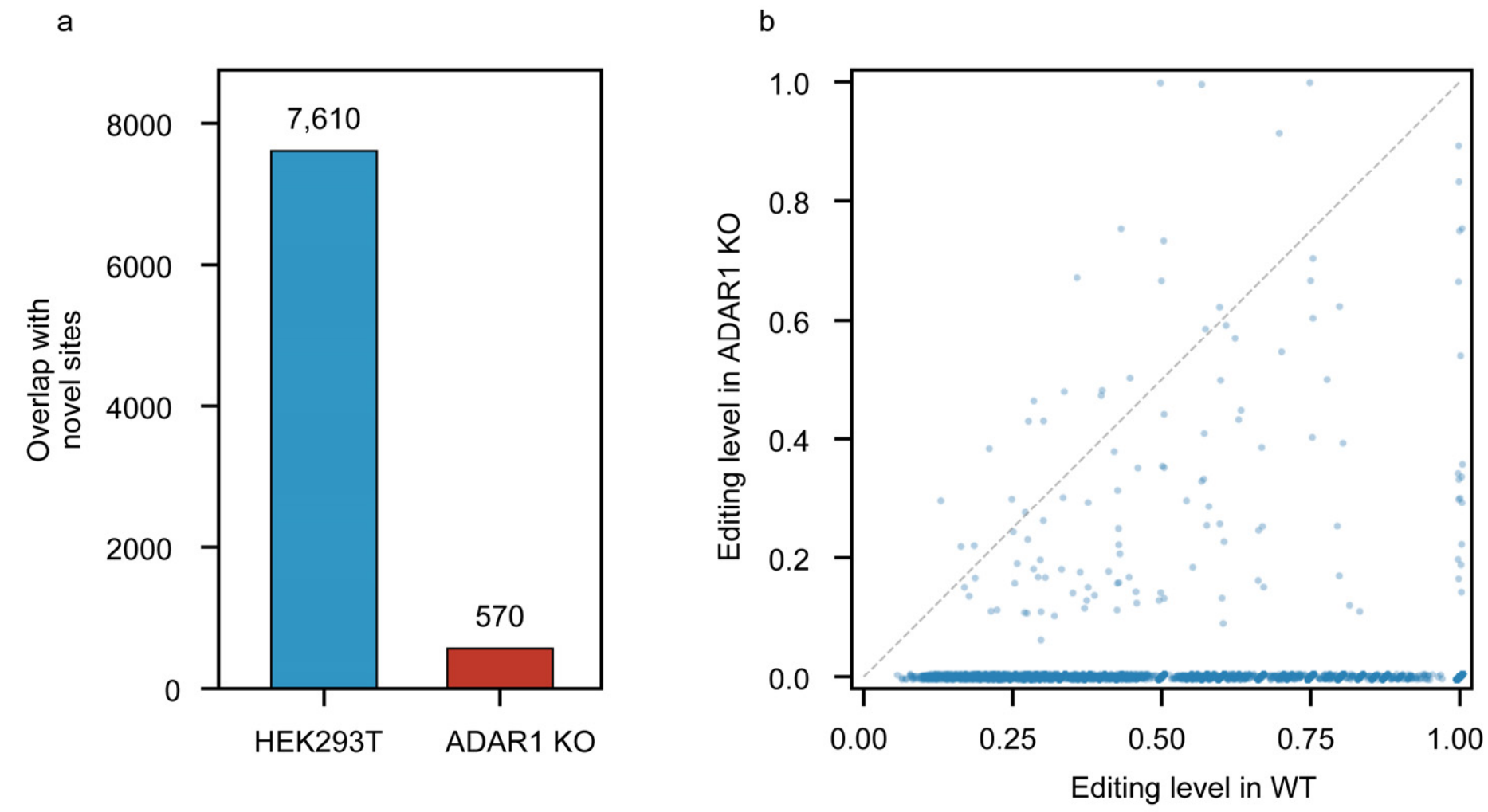

Supplementary Fig. S9 Validation of REX-seq novel sites using ADAR-null cell lines. a Number of REX-seq novel sites detected in WT (n=7,610) and ADAR1 knockout cells (n=570). **b** Scatter plot comparing editing frequencies of 7,610 novel sites between WT and ADAR1KO cells (y-axis). Dashed line represents y = x. Of the 7,610 novel sites, 7,484 (98.3%) showed markedly reduced editing in ADAR1KO cells.

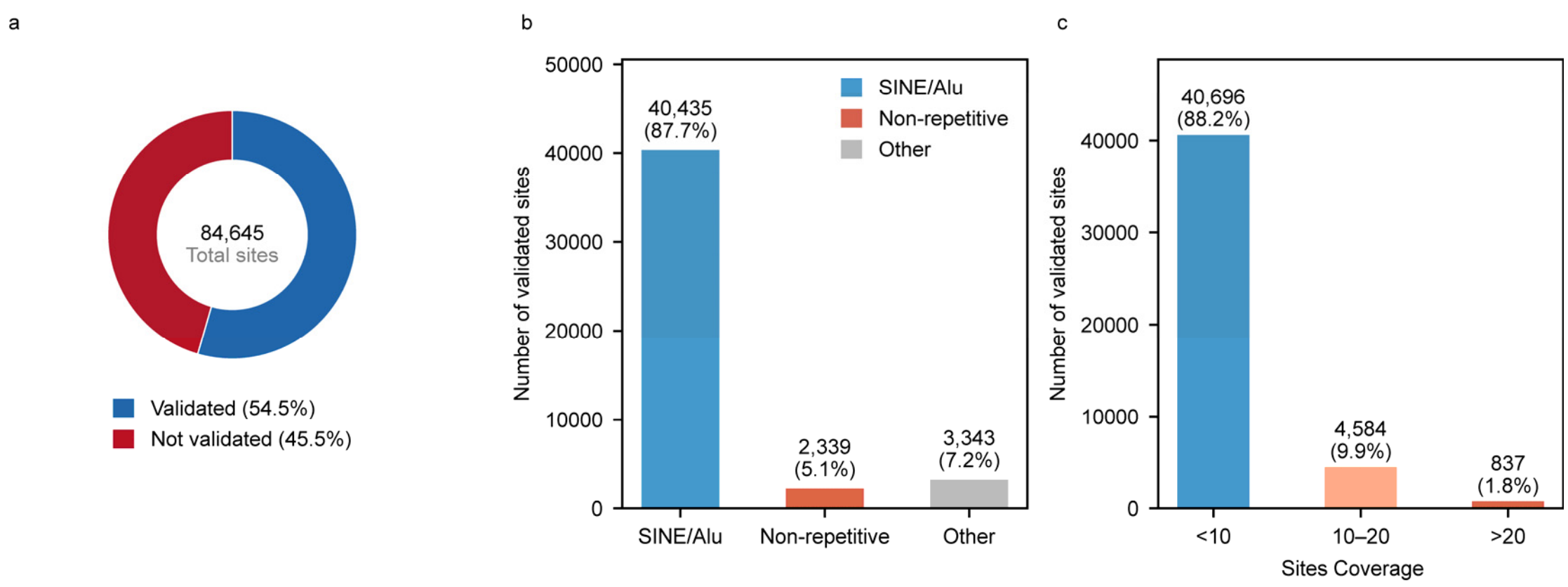

Supplementary Fig. S10 Validation of novel RNA editing sites. a Pie chart of validation status. **b** Repeat-type composition of validated sites. **c** Distribution of supporting reads among validated sites.

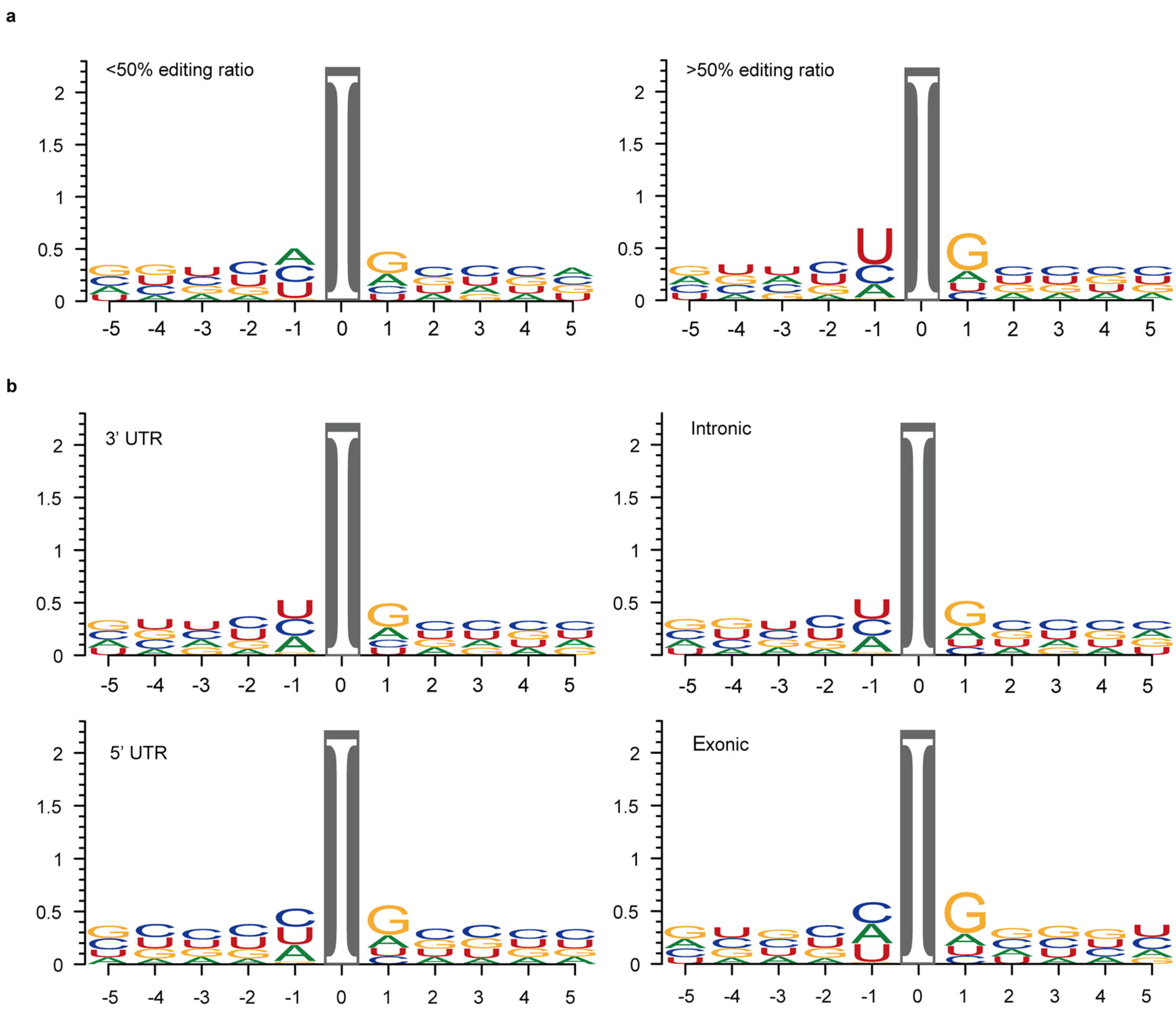

Supplementary Fig. S11 Sequence preference analysis of A-to-I editing sites. a Nucleotide distribution profiles around A-to-I editing sites (position 0) stratified by editing efficiency. Low-editing sites (<50% editing ratio) exhibit a preference hierarchy of A > C > U at the -1 position, whereas high-editing sites (>50% editing ratio) show a reversed pattern of U > C > A. **b** Sequence context analysis of novel A-to-I editing sites across genomic regions. Editing sites in 3'UTR and intronic regions share conserved upstream (U at -1) and downstream (G at +1) nucleotide biases, while 5'UTR and exonic sites exhibit distinct preferences (G at -1 for 5'UTR; A at -1 for exonic regions). All profiles are centered on the edited adenosine (position 0) with ±5 bp flanking sequence.

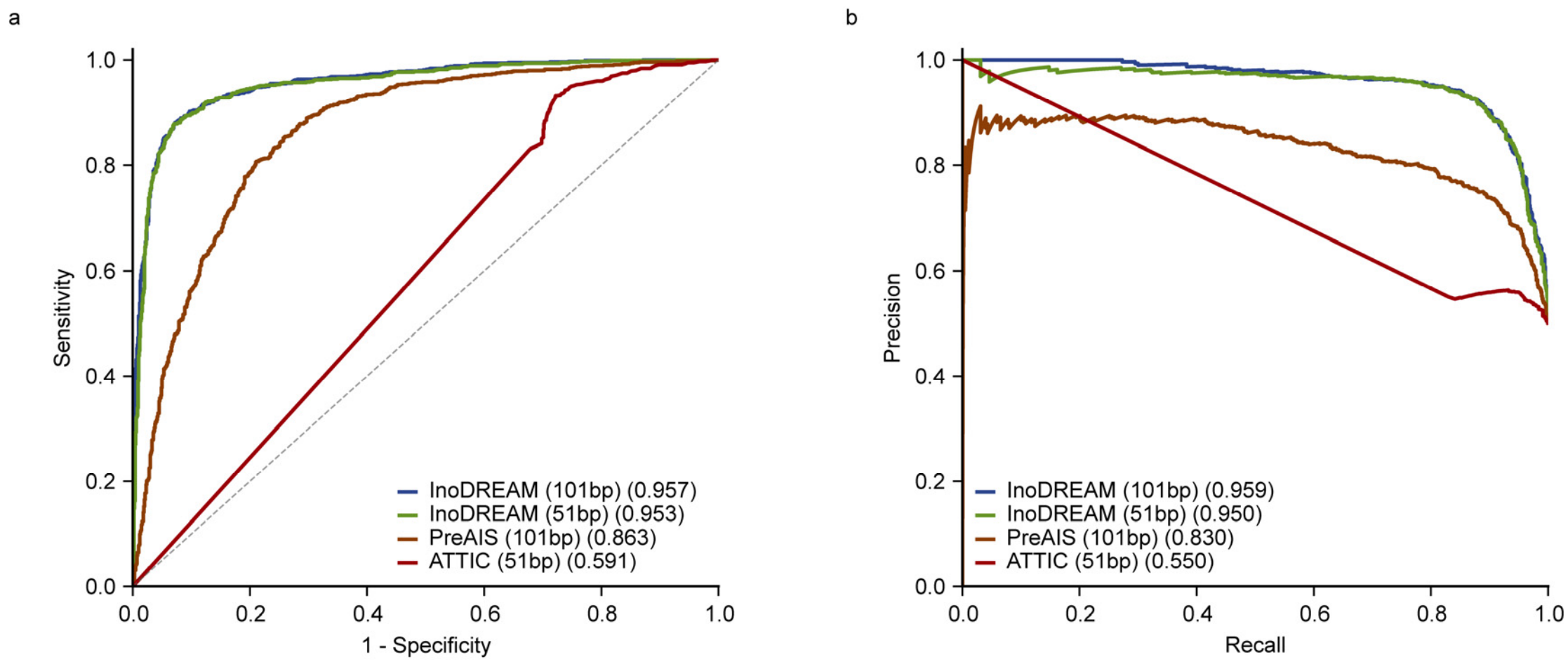

Supplementary Fig. S12 ROC and PR curves comparing A-to-I editing prediction performance. a Receiver operating characteristic curves. **b** Precision-recall curves. All models were evaluated on the same balanced subset of the REX-seq test set. InoDREAM was evaluated at two input lengths (101 bp and 51 bp) centered on the editing site. PreAIS was retrained on the same REX-seq training data as InoDREAM (n = 223,236). ATTIC was used with its pre-trained human model. AUC, area under the ROC curve; AP, average precision.

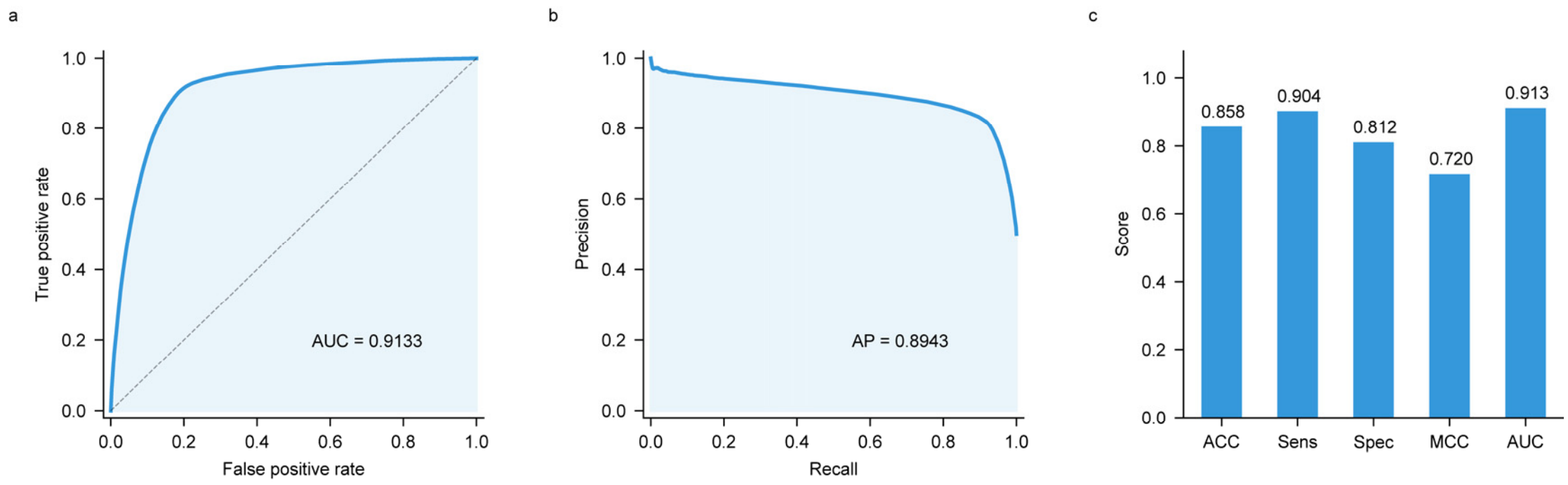

Supplementary Fig. S13 InoDREAM trained on REDIportal. The model achieved (**a**) AUC of 0.913, (**b**) AP of 0.894, and (**c**) ACC = 0.858, Sensitivity = 0.904, Specificity = 0.812, MCC = 0.720 on the held-out test set (1.27M unique editing sites), demonstrating generalizability across diverse tissues and cell types.

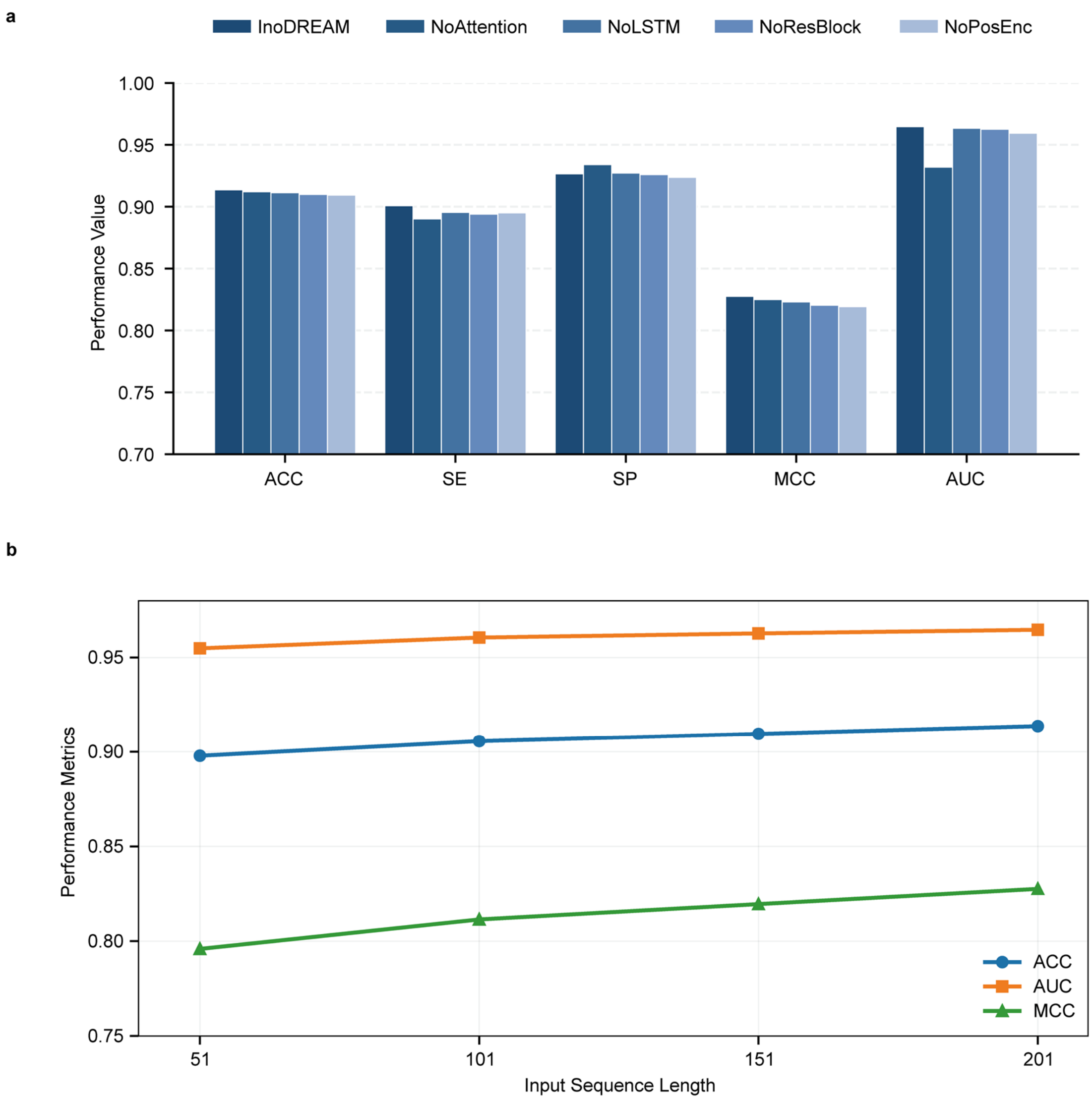

Supplementary Fig. S14 Ablation studies and sequence length optimization of InoDREAM. a Contribution analysis of InoDREAM's architectural modules via ablation experiments. The full model (InoDREAM) outperforms all module-deficient variants across most evaluation metrics, including accuracy (ACC), sensitivity (SE), Matthew's correlation coefficient (MCC), and area under the ROC curve (AUC). **b** Impact of input sequence length on model performance. Metrics including ACC, AUC, and MCC are plotted against sequence lengths of 51 bp, 101 bp, 151 bp, and 201 bp. Performance improves with increasing sequence length up to 201 bp, after which gains plateau while computational complexity increases. The optimal sequence length (201 bp) is highlighted, corresponding to ACC=0.9137, AUC=0.9647, and MCC=0.8276.

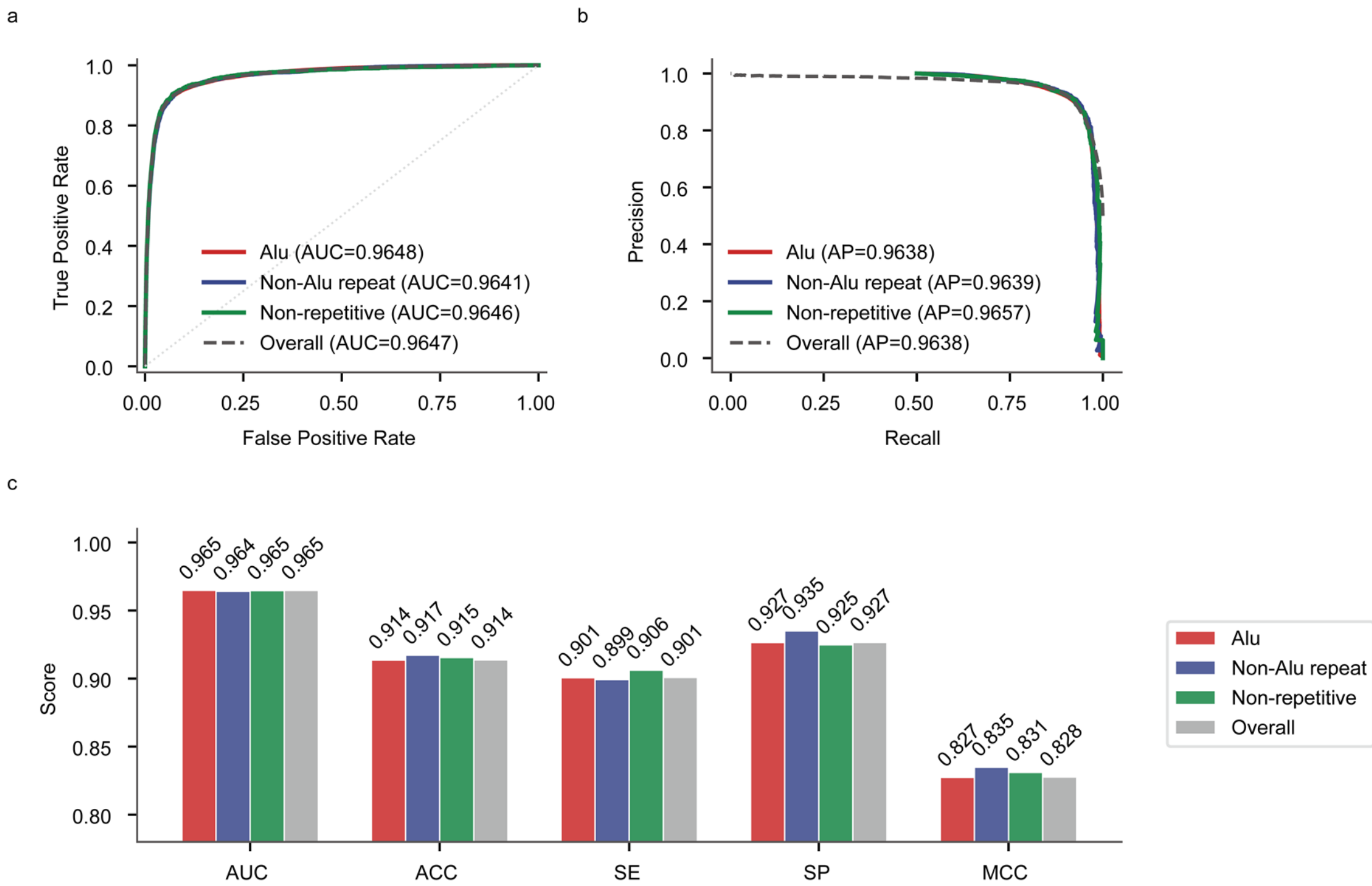

Supplementary Fig. S15 Stratified evaluation of InoDREAM performance by repeat element category. a ROC curves for each category and the overall test set. AUC values are nearly identical across Alu (0.9648), non-Alu repeats (0.9641), non-repetitive regions (0.9646), and overall (0.9647). **b** Precision-Recall curves. AP values are consistent after balanced subsampling (0.9638–0.9657). **c** Grouped bar chart comparing all five metrics across categories.

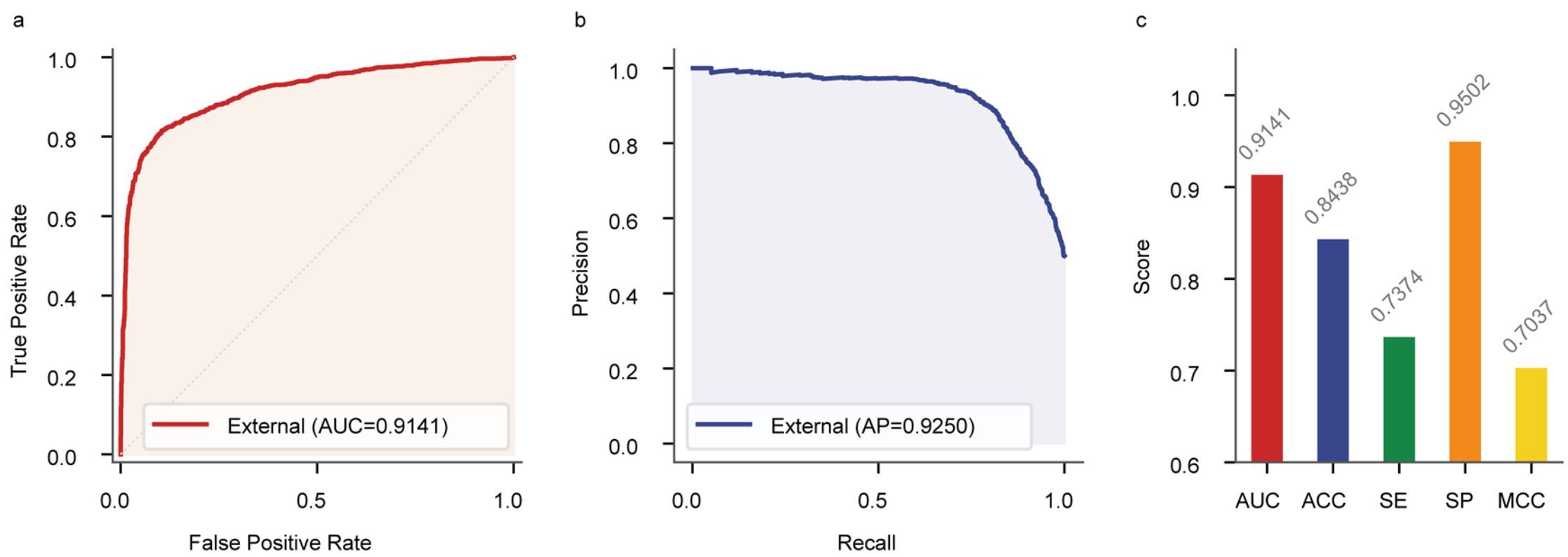

Supplementary Fig. S16 External validation of InoDREAM performance on cross-method independent test set. a ROC curve (AUC = 0.9141) and **b** Precision-Recall curve (AP = 0.9250) on an external test set of 1,546 positive and 1,546 negative sites. **c** Performance metrics including AUC, accuracy, sensitivity, specificity, and Matthews correlation coefficient (MCC).

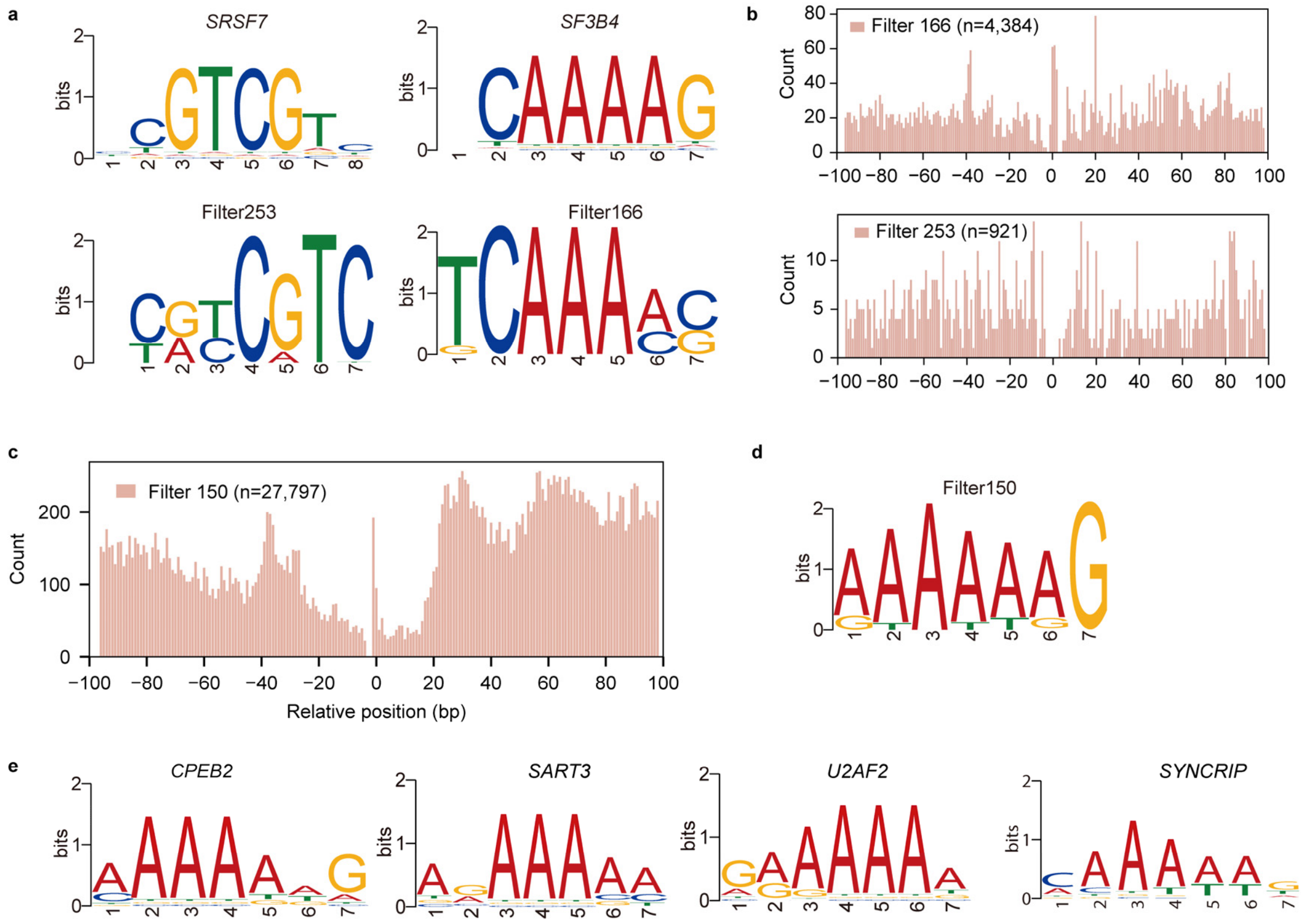

Supplementary Fig. S17 Functional element enrichment in regulatory regions flanking inosine editing sites. a Enrichment of binding motifs in proximal regions adjacent to inosine editing sites. Sequence logos depict conserved motifs identified in flanking regions, including *SRSF7*-associated motifs (Filter253), *SF3B4*-binding sites (Filter166). **b** Positional distribution of splicing factor motifs relative to editing sites. Filter166 (*SF3B4*, n=4,384) and Filter253 (*SRSF7*, n=921). Bar plots indicate the positional distribution frequency of each learned motif on sequences of 201bp. **c** Distribution of non-specific protein-binding motifs across editing site regions. Filter150 motifs (n=27,797), associated with polyadenylation signals, exhibit dispersed enrichment patterns in flanking regions, indicating broad regulatory activity. **d** Sequence logo of the non-specific polyadenylation signal-like motif (Filter150: 5'-AAAAAAAG-3'), showing conserved adenine-rich in the proximal editing site context. **e** Binding motifs of splicing-associated proteins with broad RNA-binding properties. Sequence logos illustrate characteristic motifs of *CPEB2*, *SART3*, *U2AF2*, and *SYNCRIP*, which exhibit dispersed distribution patterns across editing site regions.

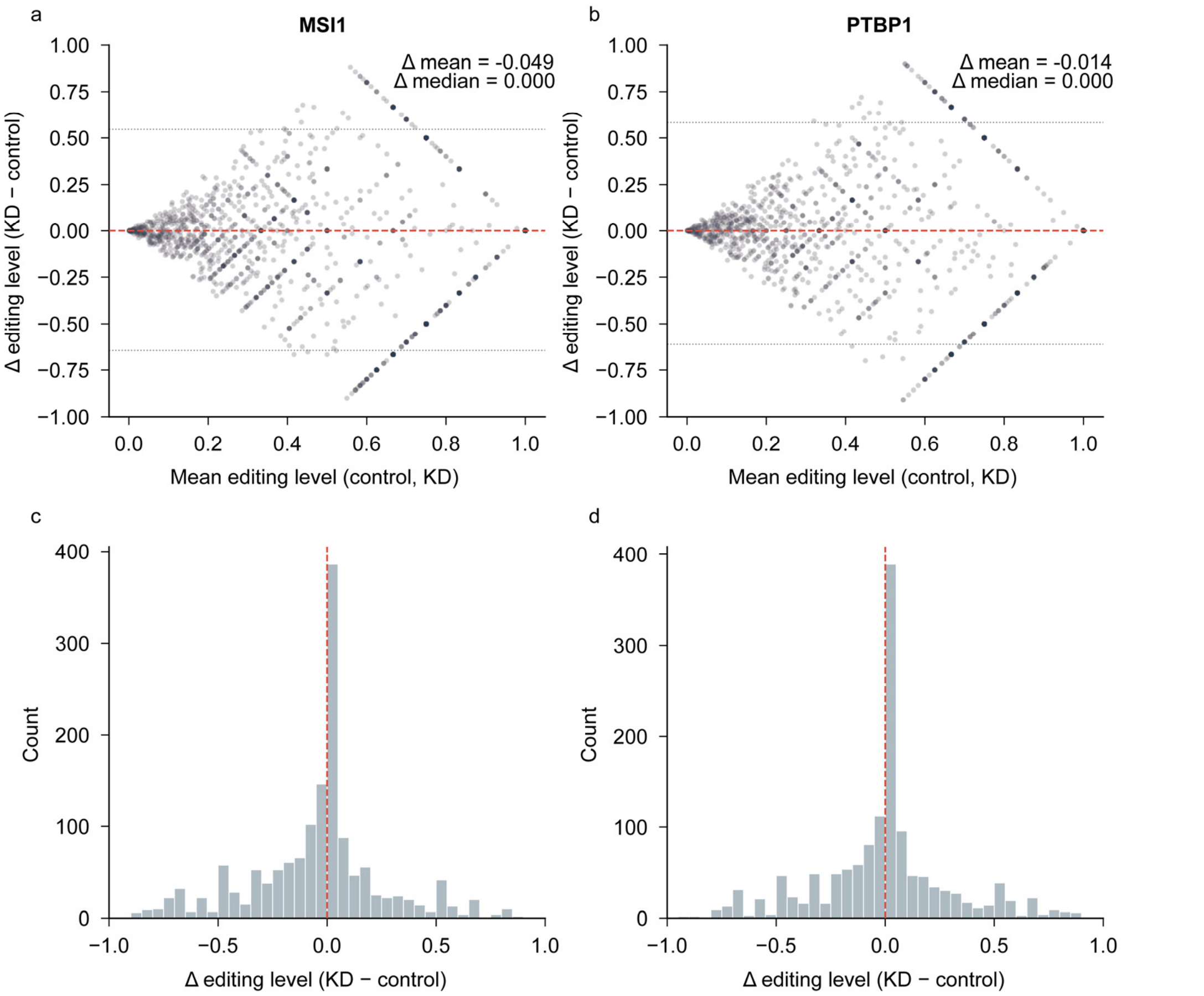

Supplementary Fig. S18 RBP knockdown at motif-associated editing sites. a Scatter plot comparing MSI1 KD, mean delta=−0.049 and control editing levels. x-axis: mean editing level of the two conditions. y-axis: change in editing level (KD minus Control). Horizontal red dashed line: delta equals 0. **b** Same as a for PTBP1 KD. Mean delta=−0.014. **c** Histogram of delta editing levels for MSI1 KD. Red dashed line: delta equals 0. **d** Histogram of delta editing levels for PTBP1 KD. Red dashed line: delta equals 0.

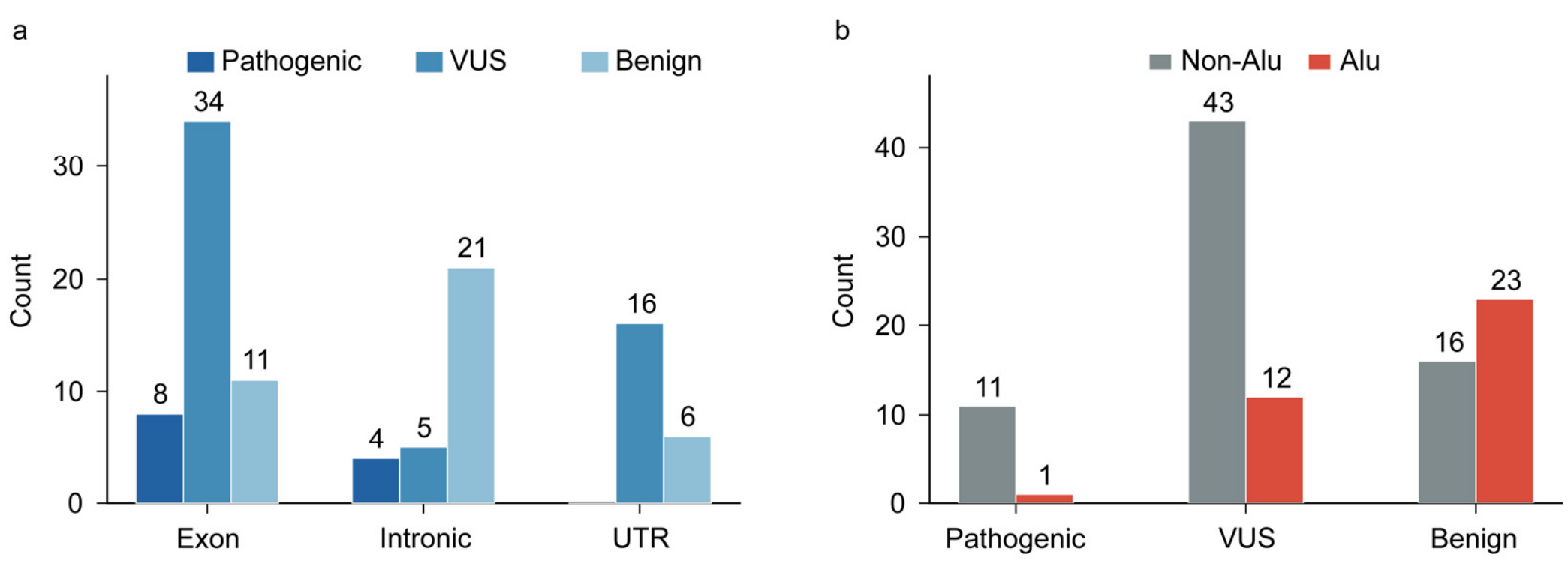

Supplementary Fig. S19 Functional classification of ClinVar-overlapping editing sites. a Grouped bar chart showing the distribution of ClinVar SNVs across functional genomic regions (exon, intronic, UTR), stratified by clinical significance. Pathogenic SNVs are predominantly in exon (67%). **b** Grouped bar chart showing the proportion of Alu versus non-Alu elements among ClinVar-affected editing sites by clinical significance.

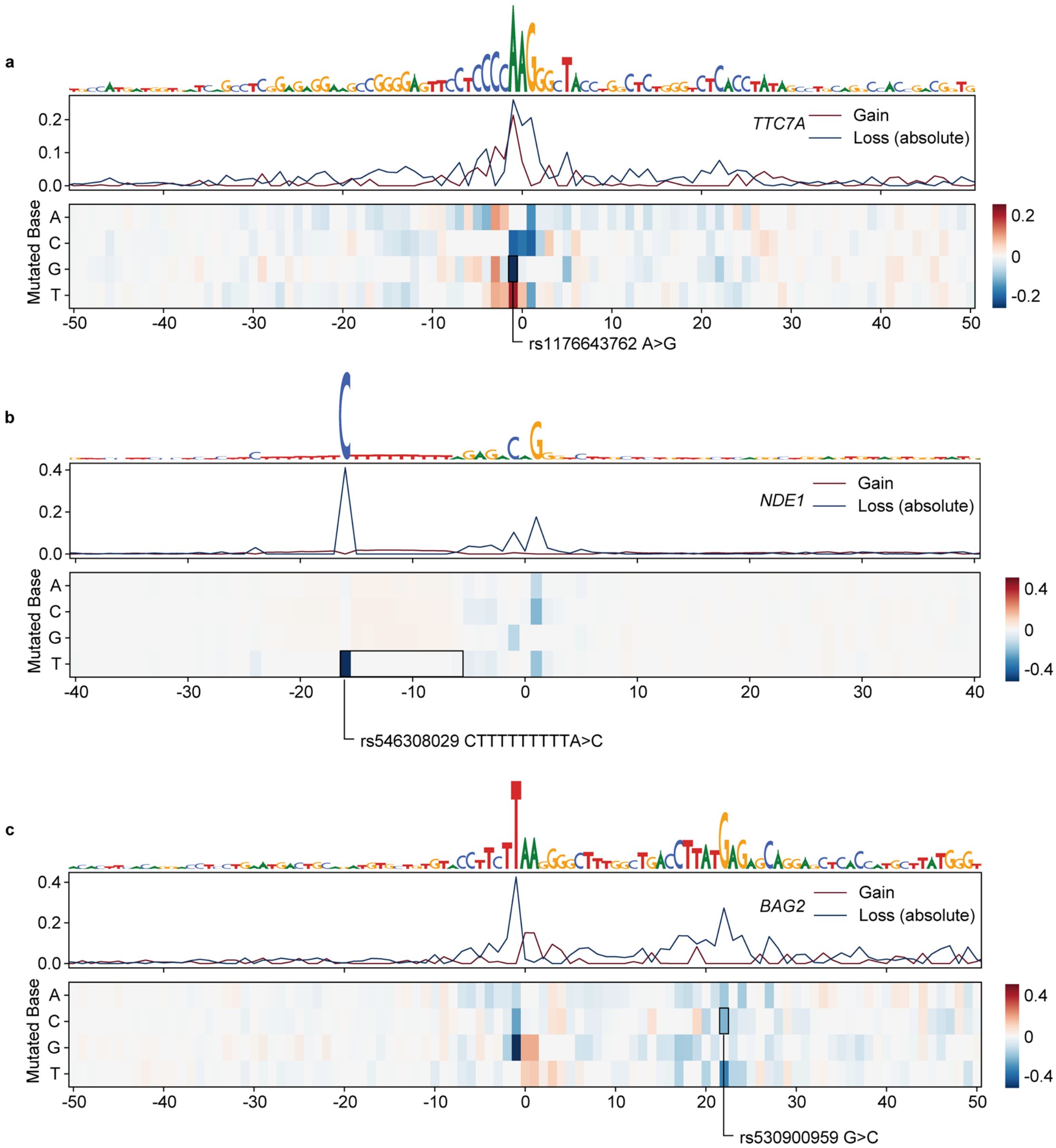

Supplementary Fig. S20 Impact of genetic variants on inosine deposition efficiency. a-c Heatmap displays the Δ Probability for each mutation event. Line plots display the minimum (loss) and maximum (gain) change among the possible substitutions. Sequence logos highlight the most significant regulatory motifs in the region. Regulatory effect of SNV in *TTC7A*, *NDE1*, *BAG2*. The rs1176643762 A>G substitution at the -1 position relative to the editing site results in a 0.26 reduction in inosine deposition (**a**). The rs546308029 indel mutation (CTTTTTTTTA>C) at the -16 position causes a 0.41 reduction in inosine deposition (**b**). The rs530900959 G>C transversion at the +22 position leads to a 0.19 decrease in inosine deposition (**c**). This variant, although classified as a variant of unknown significance (VUS) in ClinVar, localizes to a region associated with RNA stability regulation.

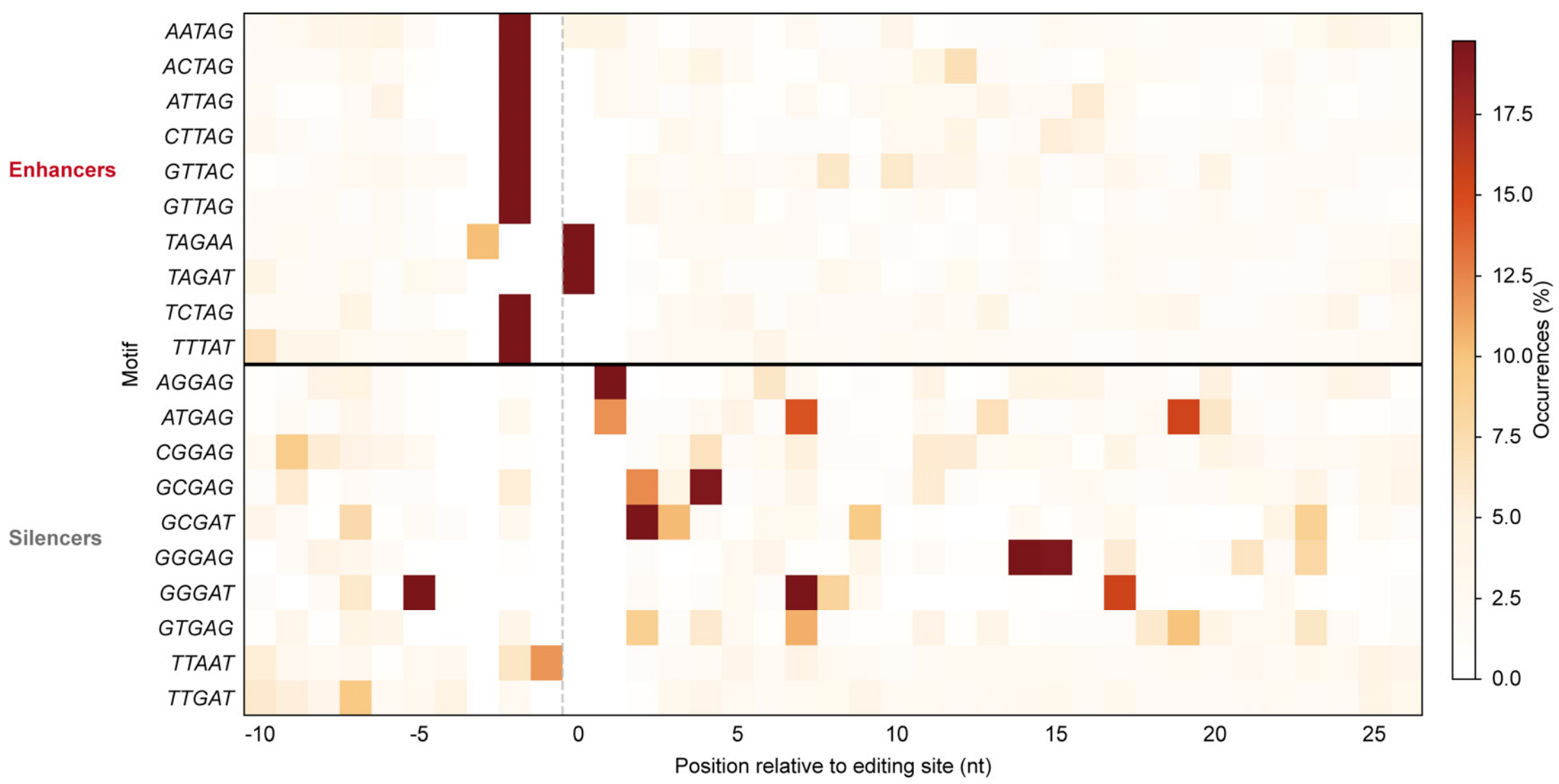

Supplementary Fig. S21 Positional distribution of enhancer and silencer motifs relative to inosine editing sites. Heatmap showing the occurrence frequency of the top 10 enhancer pentamers (partial TAG consensus) and top 10 silencer pentamers (GG/AG dinucleotides) at each position within the −10 to +30 nt window flanking inosine sites. Motif positions represent the start coordinate of each 5-mer within this region (the range is truncated at +26 nt because a 5-mer starting beyond this position would extend beyond the analysis window).

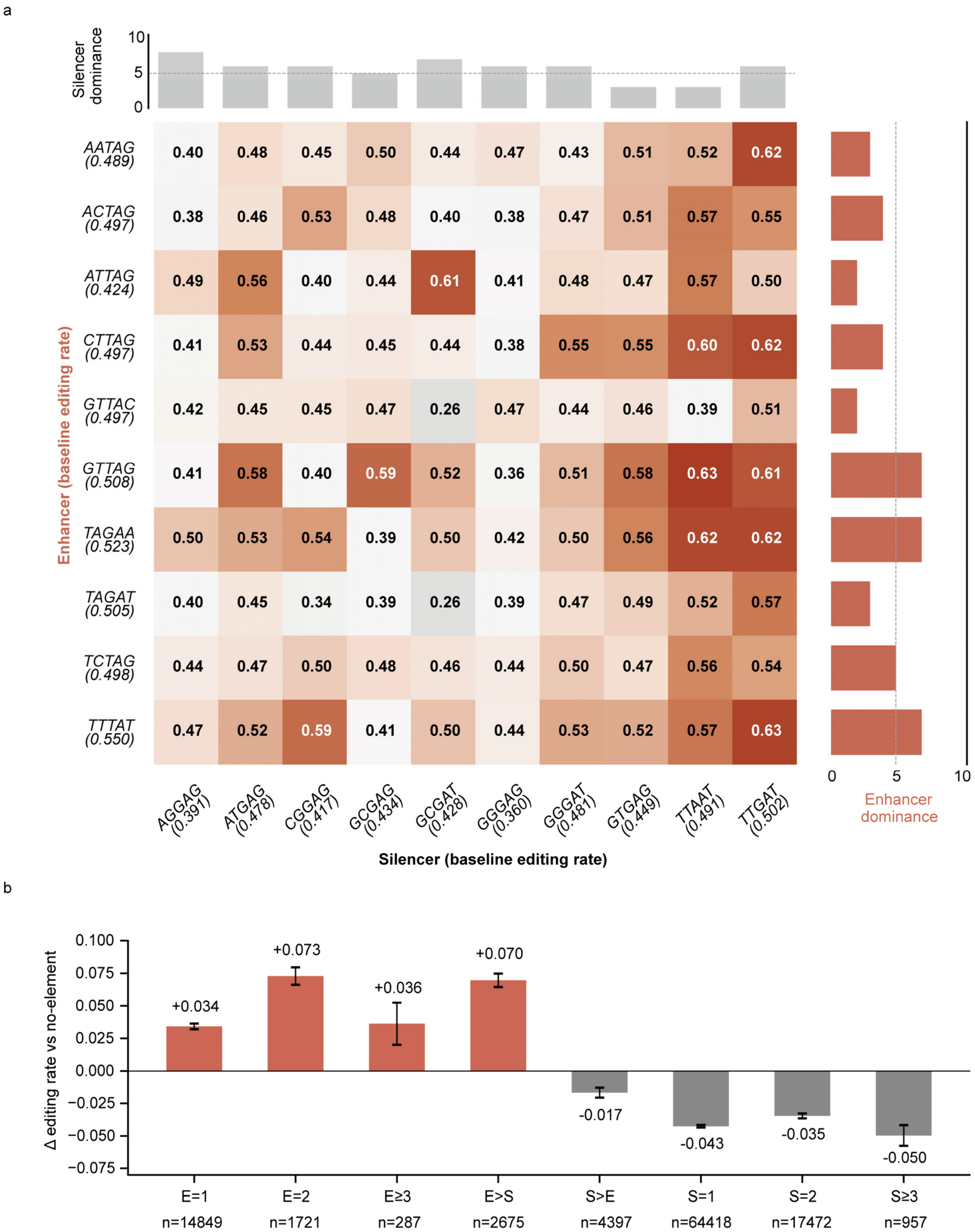

Supplementary Fig. S22 Hierarchical relationship between enhancer and silencer motifs in regulating editing efficiency. a Heatmap of mean editing rate for 7,072 sites containing exactly one enhancer (E) and one silencer (S) motif (rows = enhancers, columns = silencers). Baseline rates from sites with that motif alone (enhancer-only or silencer-only) are shown in parentheses next to each axis label. Cell color scales from gray to red by editing rate. Top and right marginal bars show the number of counterpart motifs (out of 10) where the co-occurrence rate is closer to that motif's baseline. Dashed line indicates 5/10 (no dominance). **b** Bar plot of Δ (deviation from no-element baseline of 0.455, dashed line) across eight categories: E=1, E=2, E⩾3 (no silencer), E>S (enhancer-dominant co-occurring sites), S>E (silencer-dominant co-occurring sites), S=1, S=2, S⩾3 (no enhancer). Error bars indicate SEM.

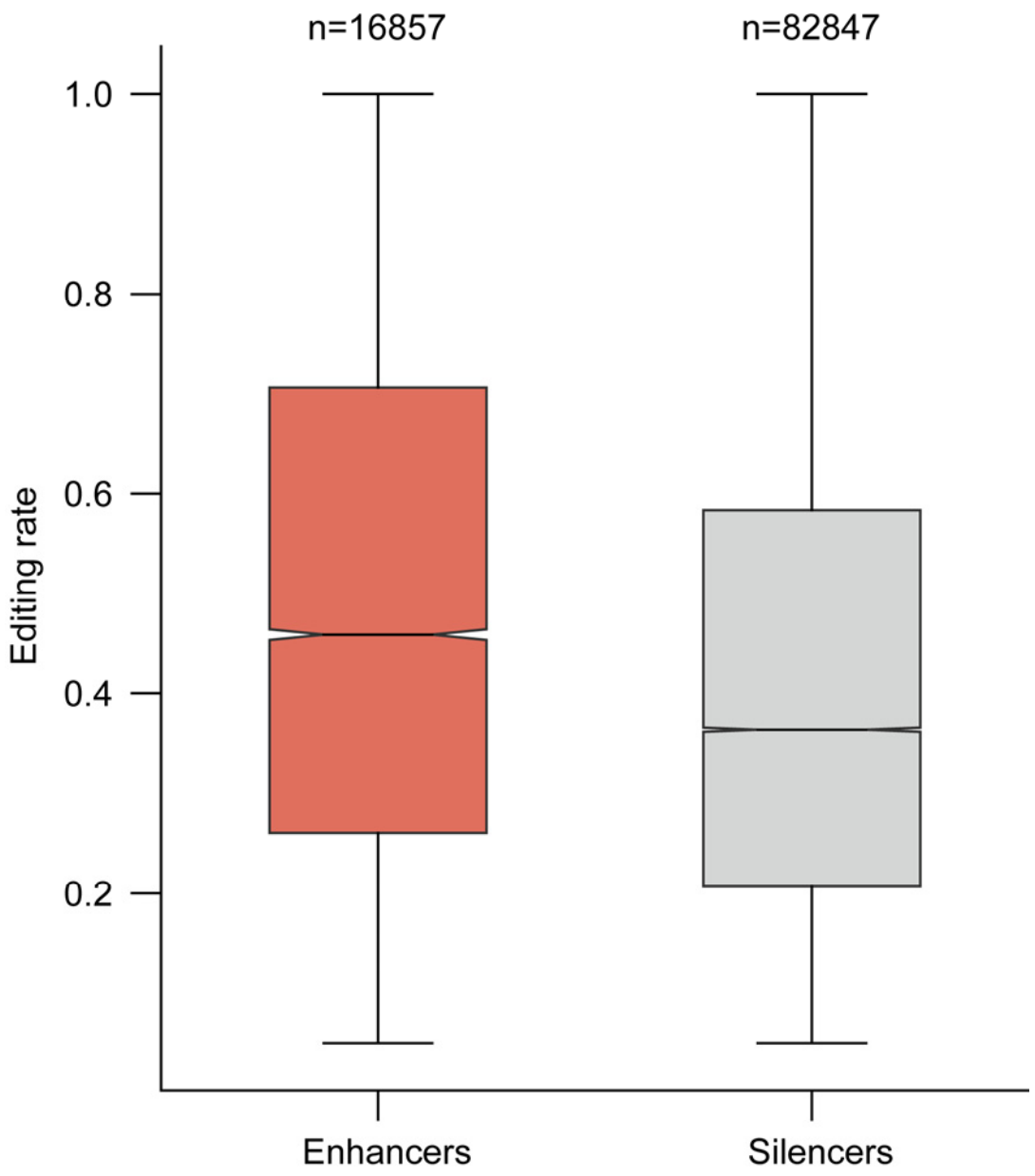

Supplementary Fig. S23 Comparison of editing rates at inosine sites adjacent to top 10 enhancers and silencers. Box plots showing the distribution of editing rates at inosine sites within the -10 nt to +30 nt region surrounding the top 10 enhancers (n=16,857) and silencers (n=82,847). Enhancers are associated with higher editing rates, whereas silencers exhibit significantly lower editing rates at adjacent inosine sites. The box represents the interquartile range (IQR), the horizontal line within the box denotes the median, and the whiskers extend to 1.5×IQR.

Supplementary Table S1. Sequence of DNA and RNA oligonucleotides used in this work

Oligonucleotides	Sequence (5′-3′)
3N-Adaptor	rAPP-NNNNNNAGATCGGAAGAGCGTCGTG-3SpC3
RT primer	ACACGACGCTCTTCCGATCT
5′adaptor	Phos-NNNNNNNNNNAGATCGGAAGAGCACACGTCTG-3SpC
RNA I（60 nt）	GUUGAUCUUGGCACGUCUGACUCGACCGUCUGU**I**GCUUGAUAGUAUCGGAUCAGUGAAUC
RNA NI（60 nt）	GUUGAUCUUGGCACGUCUGACUCGACCGUCUGU**A**GCUUGAUAGUAUCGGAUCAGUGAAUC
SgRNA 1 top	CACCG TTCTTGTAGGGTGAACACCG
SgRNA 1bottom	AAAC CGGTGTTCACCCTACAAGAA C
SgRNA 2 top	CACCG GTGCATACACTCAAGCAGTG
SgRNA 2 bottom	AAAC CACTGCTTGAGTGTATGCAC C
Asg-1-R	GCTCTATCATGTTGAACTCAC
Asg-1-F	AGACCAGAACCAGCAAGA
Asg-2-F	TCTTCTGTGATGGACTAACC
Asg-2-R	AGTGCTTATAGAGGAGATAGAG
ADAR-F1	TCCGTCTCCTGTCCAAAGAAGG
ADAR-R1	TTCTTGCTGGGAGCACTCACAC
GAPDH-F	GTCTCCTCTGACTTCAACAGCG
GAPDH-R	ACCACCCTGTTGCTGTAGCCAA
Si-MSI1-294- F	UGGAUAAAGUGCUGGCGCAAUTT
Si-MSI1-294- R	AUUGCGCCAGCACUUUAUCCATT
Si-MSI1-538- F	CACGUUUGAGAGUGAGGACAUTT
Si-MSI1-538- R	AUGUCCUCACUCUCAAACGUGTT
Si-MSI1-1123- F	CCCUUUGAUUGCCACAGCCUUTT
Si-MSI1-294- R	AAGGCUGUGGCAAUCAAAGGGTT
Si-PTBP1-833-F	CUCAACGUCAAGUACAACAAUTT
Si-PTBP1-833-R	AUUGUUGUACUUGACGUUGAGTT
Si-PTBP1-160-F	AGCAAACGGAAAUGACAGCAATT
Si-PTBP1-160-R	UUGCUGUCAUUUCCGUUUGCUTT
Si-PTBP1-415-F	CCAGCCCAUCUACAUCCAGUUTT
Si-PTBP1-415-R	AACUGGAUGUAGAUGGGCUGGTT
Si-NC- F	UUCUCCGAACGUGUCACGUUU
Si-NC- R	ACGUGACACGUUCGGAGAAUU
MSI1-F	GCTCAGCCAAAGGAGGTGATGT
MSI1-R	GCGTAGGTTGTGGCTTGGAAAC
PTBP1-F	CTCCAAGTTCGGCACAGTGTTG
PTBP1-R	CAGGCGTTGTAGATGTTCTGCC

Supplementary Table S2. Data Size of Our Data Sets

Train		Valid		Test	
postive	negative	postive	negative	postive	negative
111,618	111,618	37,205	37,205	37,205	37,205
